# Native American admixture recapitulates population-specific migration and settlement of the continental United States

**DOI:** 10.1371/journal.pgen.1008225

**Published:** 2019-09-23

**Authors:** I. King Jordan, Lavanya Rishishwar, Andrew B. Conley

**Affiliations:** 1 School of Biological Sciences, Georgia Institute of Technology, Atlanta, Georgia, United States of America; 2 IHRC-Georgia Tech Applied Bioinformatics Laboratory, Atlanta, Georgia, United States of America; 3 PanAmerican Bioinformatics Institute, Cali, Valle del Cauca, Colombia; University of Pennsylvania, UNITED STATES

## Abstract

European and African descendants settled the continental US during the 17^th^-19^th^ centuries, coming into contact with established Native American populations. The resulting admixture among these groups yielded a significant reservoir of Native American ancestry in the modern US population. We analyzed the patterns of Native American admixture seen for the three largest genetic ancestry groups in the US population: African descendants, Western European descendants, and Spanish descendants. The three groups show distinct Native American ancestry profiles, which are indicative of their historical patterns of migration and settlement across the country. Native American ancestry in the modern African descendant population does not coincide with local geography, instead forming a single group with origins in the southeastern US, consistent with the Great Migration of the early 20^th^ century. Western European descendants show Native American ancestry that tracks their geographic origins across the US, indicative of ongoing contact during westward expansion, and Native American ancestry can resolve Spanish descendant individuals into distinct local groups formed by more recent migration from Mexico and Puerto Rico. We found an anomalous pattern of Native American ancestry from the US southwest, which most likely corresponds to the *Nuevomexicano* descendants of early Spanish settlers to the region. We addressed a number of controversies surrounding this population, including the extent of Sephardic Jewish ancestry. *Nuevomexicanos* are less admixed than nearby Mexican-American individuals, with more European and less Native American and African ancestry, and while they do show demonstrable Sephardic Jewish ancestry, the fraction is no greater than seen for other New World Spanish descendant populations.

## Author summary

The post-Colombian settling of North America brought African, European, and Native American populations into close proximity for the first time. The inevitable admixture among these groups resulted a reservoir of Native American ancestry in modern US populations, outside of traditional Native American groups. Here we characterize that Native American ancestry in a geographically diverse set of African descendant, Western European descendant, and Spanish descendant populations. We show that Native American ancestry in the US population is not monomorphic, strongly related to geography, and suggestive of frequent historical admixture between European settlers and local Native American groups. We also show the presence of a unique, admixed Spanish population in the Southwestern US, the modern *Nuevomexicanos*, that is distinct from other Spanish descendant groups.

## Introduction

Native Americans inhabited the area that now makes up the continental US for thousands of years prior to the arrival of the first European settlers. The ancestors of modern Native Americans are thought have arrived in the Americas from Asia, by way of the Bering Strait, in several successive waves of migration [1]. The current model, based on archaeology and comparative genomic studies, holds that the earliest ancestors of Native Americans arrived in the Americas ~23,000 years ago [2]. The earliest evidence for Native Americans in the continental US dates to ~14,000 years ago [3]. The much later arrival of Europeans in the Americas, followed shortly thereafter by Africans who were brought by force via the trans-Atlantic slave trade, had a drastic effect on the demographic makeup of the region. Native American population numbers declined rapidly in the face of continuous immigration, settlement, and conflict, and as a result the modern US population is made up mainly of descendants of European and African immigrants.

Europeans arrived in the Americas more than 20,000 years after the first Native Americans. The first European settlers to reach the continental US were Spaniards led by the conquistador Ponce de León, who claimed Florida for the Spanish crown in 1513 [4]. British settlers arrived more than 70 years later, initially establishing the ill-fated colony of Roanoke in 1585 and later the permanent settlement of Jamestown in 1607 [5]. An estimated 400,000 British had migrated to the US by the end of the 17^th^ century. The first Africans were brought to Jamestown in 1619 by Dutch pirates who traded them to the British settlers as indentured servants [6]. The social status of Africans in the US changed quickly, with slavery first legally sanctioned by 1640. The trans-Atlantic slave trade would eventually bring ~400,000 enslaved Africans to the continental US [7].

The arrival of Europeans and Africans in the Americas, and the conflict that followed, would prove to be catastrophic for the indigenous population. It has been estimated that 10–100 million Native Americans may have died in the first 150 years after Columbus’ arrival in the New World, amounting to a 95% reduction in the population [8]. This massive Native American population decline is mainly attributed to the introduction of European and African endemic infectious diseases–*e*.*g*. malaria, measles, and smallpox–for which the indigenous population had little or no immune defense [8, 9].

The story of conflict between Native Americans and European settlers and enslaved Africans, along with the devastating consequences for the indigenous population, is by now well-known. However, there is another, perhaps less appreciated, aspect of the encounter between these population groups that has also had profound consequences for the genetic demography of the Americas. Here, we are referring to the process of genetic admixture, whereby individuals from previously isolated population groups reproduce, resulting in the novel combination of ancestry-specific haplotypes within individual genomes. Admixture has been a fundamental feature of human evolution and migration [10]. Whenever previously isolated human populations meet, no matter what the circumstances, they mix and give rise to individuals with a mosaic of different genetic ancestries.

As European and African descendants settled the continental US, they inevitably came into contact with established Native American populations resulting in admixture and the introduction of Native American genomic sequence into the expanding US population. Accordingly, the genomes of European and African descendants in the US are expected to contain some fraction of Native American ancestry. In other words, a significant reservoir of Native American ancestry currently exists outside of recognized indigenous communities. In this study, we ask how the historical processes of migration and settlement affected the distribution of Native American admixture across the continental US (S1 Fig). We address this question for the three largest genetic ancestry groups in the modern US population: African descendants (AD), Western European descendants (WD), and Spanish descendants (SD).

## Results

### Genetic ancestry groups in the US

The first aim of our study was to characterize the major genetic ancestry groups for the continental US based on observable patterns of ancestry and admixture seen for the 15,620 individuals from the Health and Retirement Study (HRS) analyzed here. The Health and Retirement Study data is sponsored by the National Institute on Aging (grant number U01AG009740) and is conducted by the University of Michigan. Having defined the US genetic ancestry groups, we then considered the distribution of Native American admixture within and between ancestry groups and among geographic regions. We provide a detailed description (S1 Text), along with supporting results (S3–S8 Figs, S2 and S3 Tables), of how we defined the three main US ancestry groups–African descendants, Western European descendants, and Spanish descendants–in the Supplementary Material.

**Fig 1:**
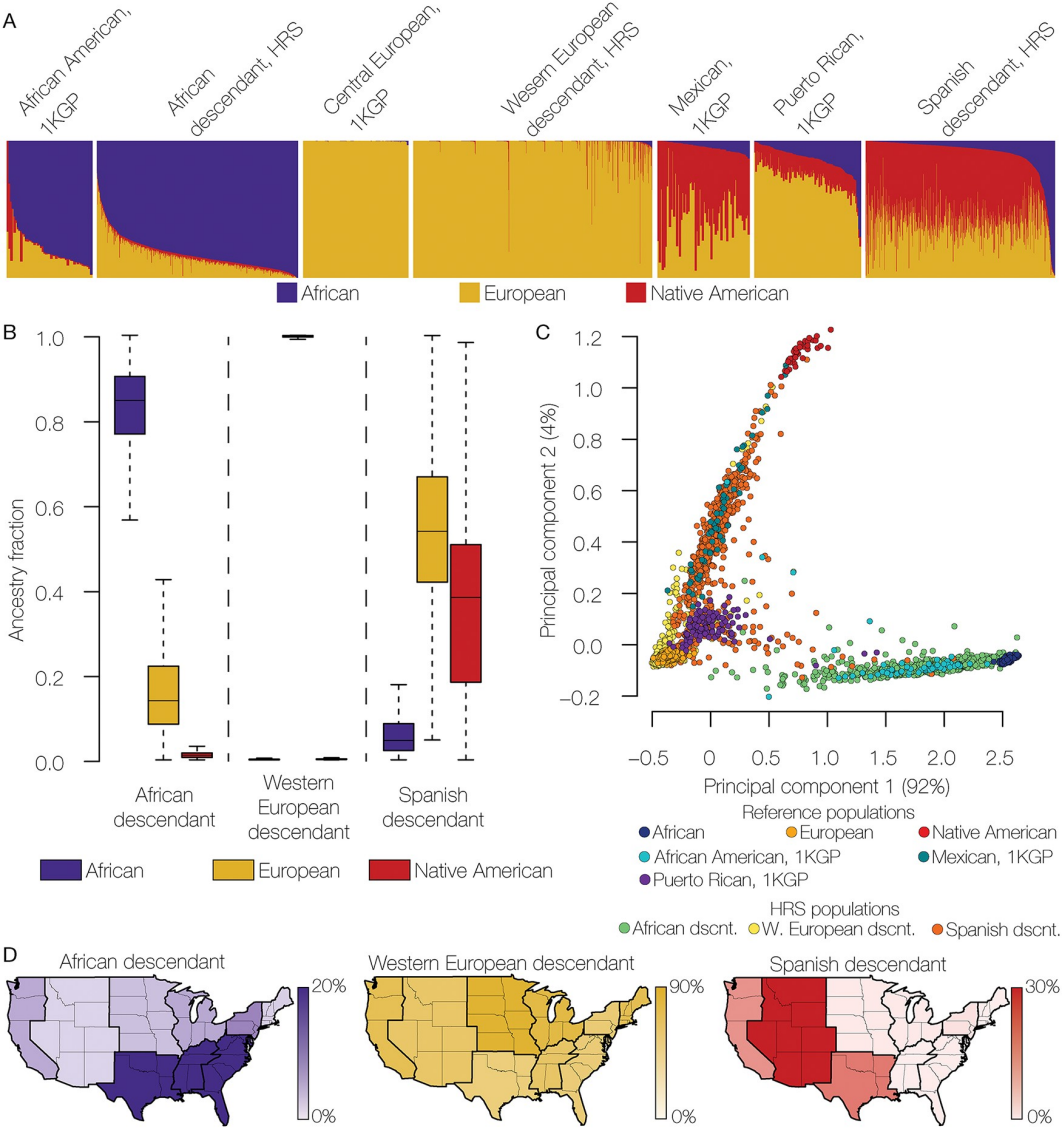
Genetic ancestry groups in the modern US population. (A) ADMIXTURE plot (*K* = 3) showing the African (blue), European (gold), and Native American (red) ancestry components for individuals from different US population groups. Data are from the 1000 Genomes Project (1KGP) and the Health and Retirement Study (HRS). (B) Distributions of African, European, and Native American ancestry fractions for the three main US genetic ancestry groups defined here: African descendant, Wester European descendant, and Spanish descendant. (C) Principal components analysis (PCA) plot showing the relationships among individuals from reference populations and individuals from the HRS dataset corresponding to the three US genetic ancestry groups. (D) Percentages of individuals from each of the three US genetic ancestry groups are shown for the nine census regions in continental US.

The ancestry distribution of HRS individuals among the three largest US genetic ancestry groups is shown in Fig 1. Visual inspection of the continental ancestry fractions seen for members of the three groups supports our approach to genetic ancestry-based classification (Fig 1A). For example, the majority of Spanish descendant individuals show substantially higher Native American ancestry compared to Western European descendants (Fig 1A); the median Native American ancestry for the Spanish descendant group is 38% compared to 0.1% for the Western European descendant group (Fig 1B). In addition, individuals from the Spanish descendant group cluster tightly with the Mexican reference population from the 1KGP, along the second axis between the European and Native American populations in the principal components analysis (PCA) plot of the pairwise genome distances (Fig 1C). It is important to note that we did not use Native American ancestry for the purposes of classification. Rather, European ancestry alone was sufficient to recapitulate known levels of Native American ancestry for Spanish descendants.

Individuals from the African descendant group show medians of 85% African ancestry, 14% European ancestry, and 1% Native American ancestry (Fig 1B). Most of these individuals group along the first PCA axis separating the African and European reference populations. In contrast to the admixed Spanish and African descendant groups, Western European descendants show extremely low levels of admixture with non-European populations, with a median value of 99.8% European ancestry. Given their relatively low numbers (S2 Fig), as well as their relatively late historical arrival in the continental US, we did not consider Asian descendants further in this study.

Individuals assigned to the three main genetic ancestry groups show distinct geographic distributions across the continental US, which are largely consistent with demographic data for the country. The proportion of African descendants is highest in the three southern census regions, Western European descendants in the two north central regions, and Spanish descendants in the Mountain census region, which includes Arizona and New Mexico (Fig 1D).

### Sex-biased admixture in US genetic ancestry groups

**Fig 2:**
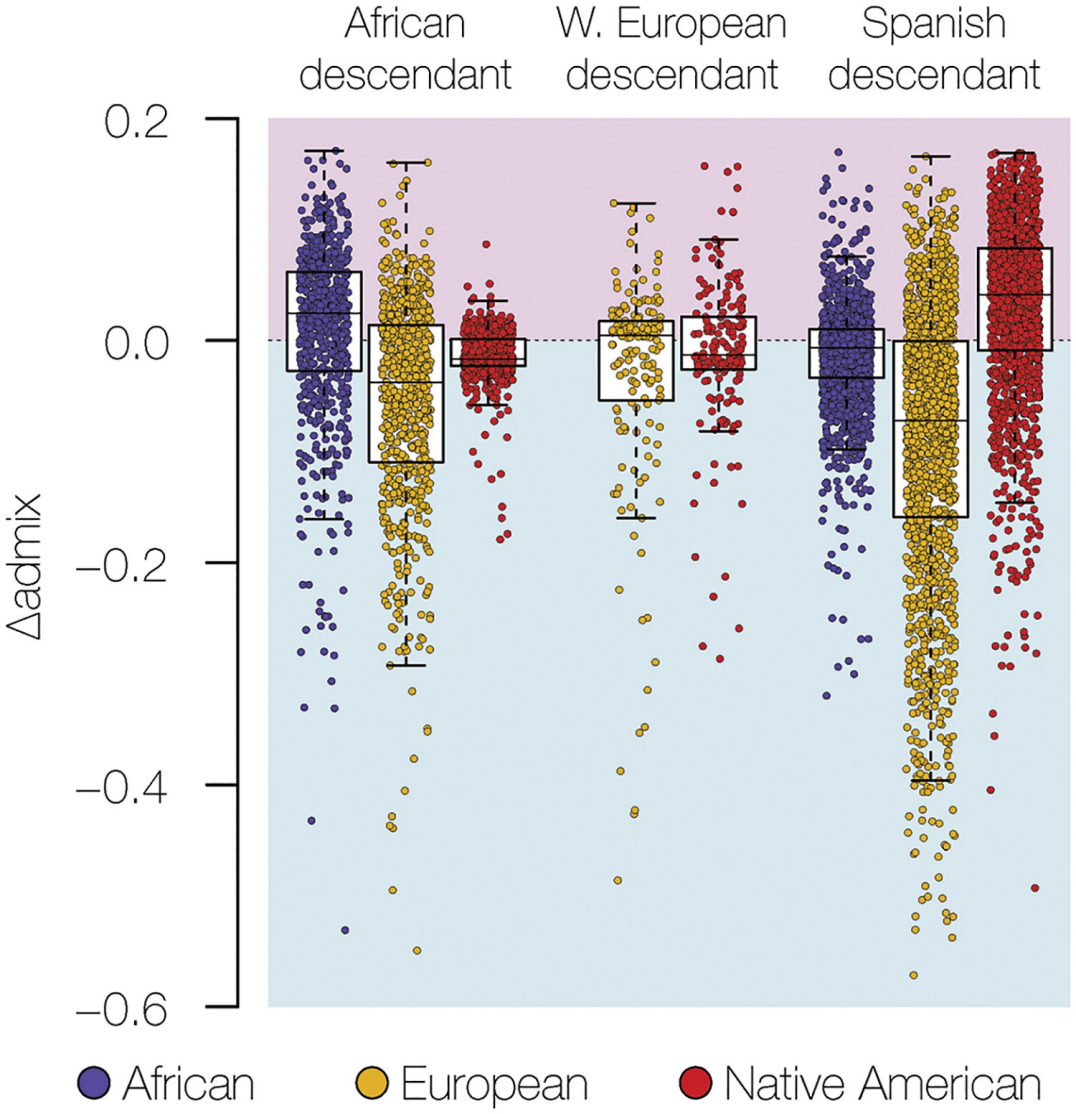
Sex-biased admixture in US genetic ancestry groups. Normalized differences between X chromosome ancestry fractions and autosomal ancestry fractions (*ΔAdmix*) are shown on the y-axis. *ΔAdmix* values are shown for each ancestry component–African (blue), European (gold), and Native American (red)–in each individual genome. *ΔAdmix* values above zero (pink) indicate female-biased admixture, and values below zero (blue) indicate male-biased admixture.

We compared the patterns and extent of sex-biased admixture among the three US genetic ancestry groups by comparing the continental ancestry fractions–African, European, and Native American–seen for the X chromosomes versus the autosomes. For any given ancestry component, a relative excess of X chromosome ancestry is indicative of female-biased admixture, whereas an excess of autosomal ancestry reflects male-biased admixture [11]. This was only done for admixed individuals that had two or more continental ancestry fractions at >1.5% of the overall ancestry. Almost all individuals from the African and Spanish descendant groups met this criterion, but only a small minority of Western European descendant individuals with Native American admixture did. African and Spanish descendant groups showed marked patterns of sex-biased admixture, whereas the Western European descendants did not show any appreciable evidence of sex-biased admixture (Fig 2). The strongest pattern of sex-biased admixture was seen for Spanish descendants, with female-biased Native American admixture and male-biased European admixture. African descendants show female-biased African ancestry and male-biased European ancestry.

### Native American ancestry distribution across the US

For each US genetic ancestry group, we considered three distinct characteristics of Native American ancestry across the continental US: (1) the relative levels of Native American ancestry genome-wide, (2) the patterns of Native American allele frequencies, and (3) the phylogenetic relationships among US populations based on their Native American ancestry.

**Fig 3:**
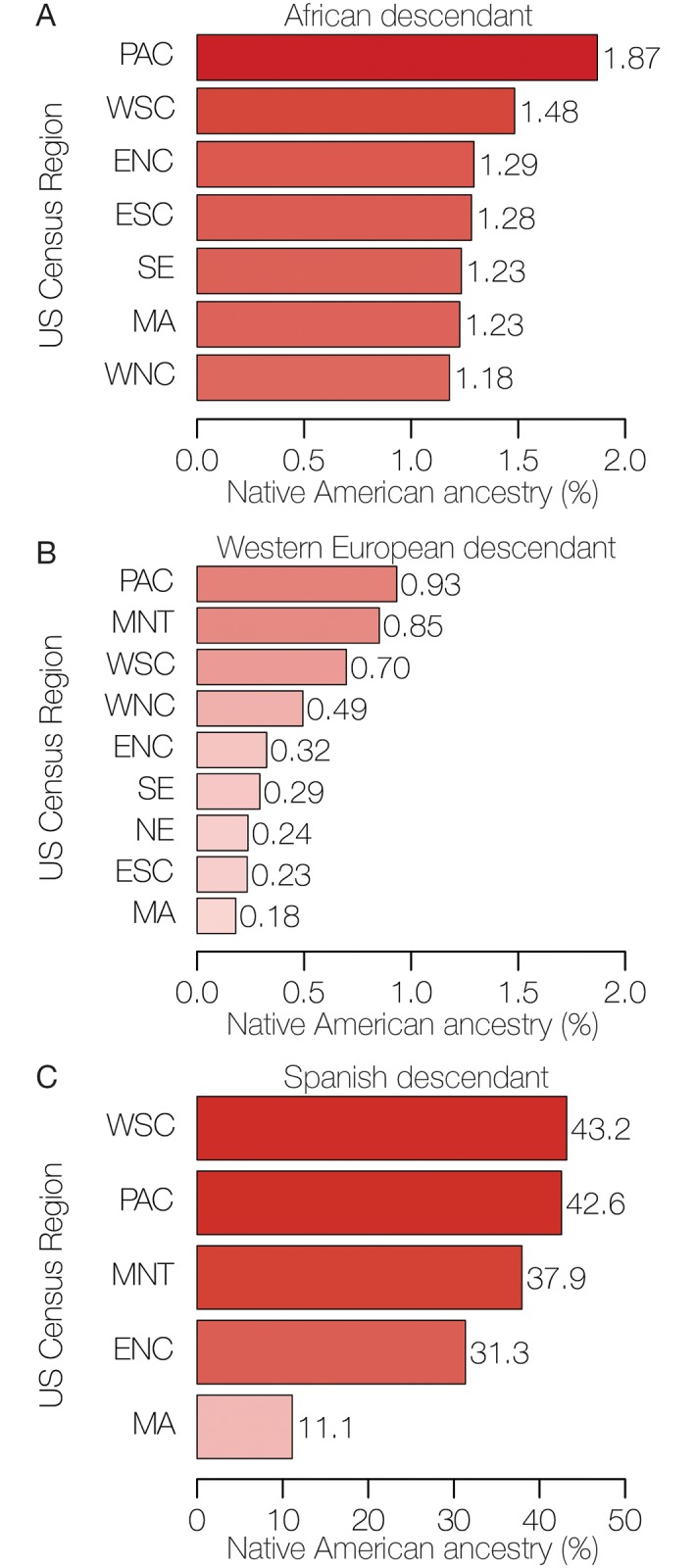
Native American ancestry percentages in the modern US population. The average percentages of Native American ancestry are shown for the three US genetic ancestry groups across the nine geographic census regions (S1 Fig) for (A) African descendant, (B) Western European descendant, and (C) Spanish descendant populations. Data for census regions with less than five individuals for any ancestry group are considered unreliable and are not shown.

As we showed previously, overall Native American ancestry is highest for the Spanish descendant group (median 38%, SD = 20.1), followed by the African descendant (1%, SD = 4.4) and Western European descendant groups (0.1%, SD = 2.7) (Fig 1B). Among all three ancestry groups, the highest levels of Native American ancestry are seen for the West-South-Central (WSC; including Texas), Pacific (PAC; including California), and Mountain (MNT; including Arizona and New Mexico) census regions (Fig 3). Native American ancestry levels show the highest variability among regions for the Spanish descendant group (coefficient of variation [c.v.] = 1.08), followed by the Western European descendant (c.v. = 0.65) and African descendant (c.v. = 0.60) groups.

We characterized the ancestry-specific and genome-wide haplotype heterozygosity (HH) for each of the admixed populations to interrogate how admixture has affected the diversity of the populations (S9 Fig, S4 Table). Where present, the African-specific HH was the highest for each population and the Native American HH was the lowest, consistent with previous observations of present day populations [12]. The genome-wide HH was significantly higher than any ancestry-specific HH for the African descendant populations, consistent with the introduction of novel haplotypes into the already diverse African background. Spanish descendant genome-wide HH was significantly higher than both the European and Native American-specific HH, but lower than African, which contributes only a small fraction of the total ancestry in the present-day Spanish descendant populations. The Western European descendant populations show a relatively very small amount of Native American ancestry; accordingly, the genome-wide HH shows no significant difference from the European HH, but is nevertheless higher than the Native American HH.

**Fig 4:**
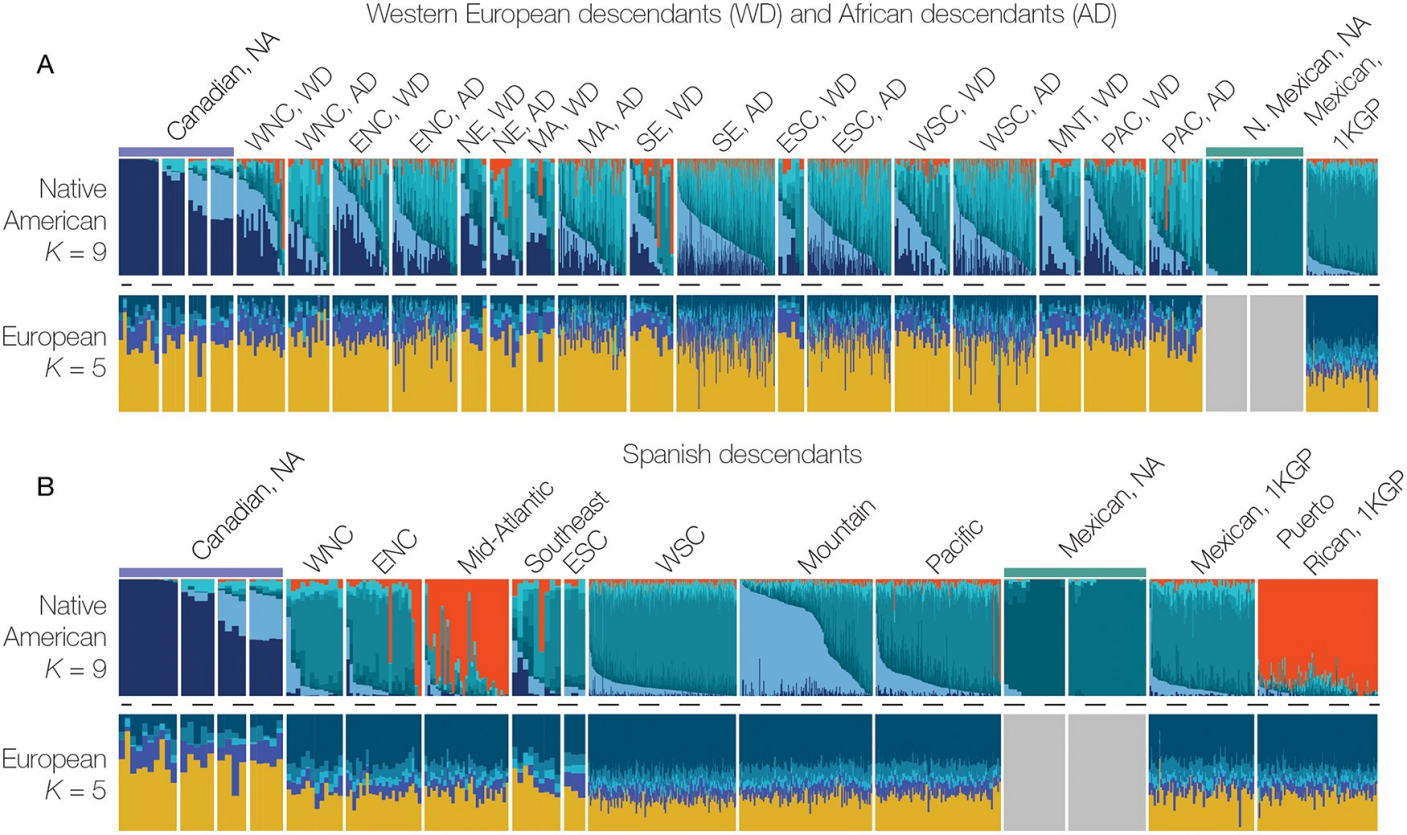
Native American and European ancestry profiles for US ancestry groups. Native American (*K* = 9) and European (*K* = 5) ancestry-specific ADMXITURE plots are shown for the Western European descendant (WD) and African descendant (AD) groups combined (A) and for the Spanish descendant group (B). The individual panels shown correspond to Native American (NA) reference populations, 1000 Genomes Project reference populations and the HRS data from the different US census regions (S1 Fig).

We measured the patterns of Native American allele frequencies across the continental US using ADMIXTURE analysis of Native American haplotypes for individuals from the three ancestry groups. Visualization of the ancestry vectors produced by ADMIXTURE shows that the African and Western European descendant groups have patterns that are similar to each other (Fig 4A, top panel; S10 Fig) and distinct from the patterns seen for the Spanish descendant group (Fig 4B, top panel; S11 Fig). Comparing the ADMIXTURE vectors of these two population groups to those of the Spanish descendant populations shows that African descendant and Western European descendant populations are significantly closer to each other than either is to the Spanish descendant populations (S1 Text, S12 and S13 Figs). Furthermore, the African descendant and Western European descendant groups show ancestry patterns that are intermediate to the Canadian and Northern Mexican Native American reference populations, whereas the Spanish descendant group shows Native American ancestry patterns that are more similar to the Mexican reference population, Mexican Native American populations, or the admixed Puerto Rican population. This is consistent with the fact that we use Native American reference populations from outside the US to identify Native American haplotypes in US population groups. There is substantial regional variation in Native American ancestry seen in the Spanish descendant group, with characteristically Mexican patterns seen in the Pacific (PAC) and West South-Central (WSC) regions and a strongly Puerto Rican pattern in the Mid-Atlantic (MA) region. At *K* = 9, ADMIXTURE is able to resolve the two Northern Mexican Native American reference populations, as well as reveal a unique ancestry in the Spanish descendant population from the Mountain (MNT) region. This population shows a distinct pattern of Native American ancestry under any *K* (S11 Fig), which we explore in more detail in the following section.

**Fig 5:**
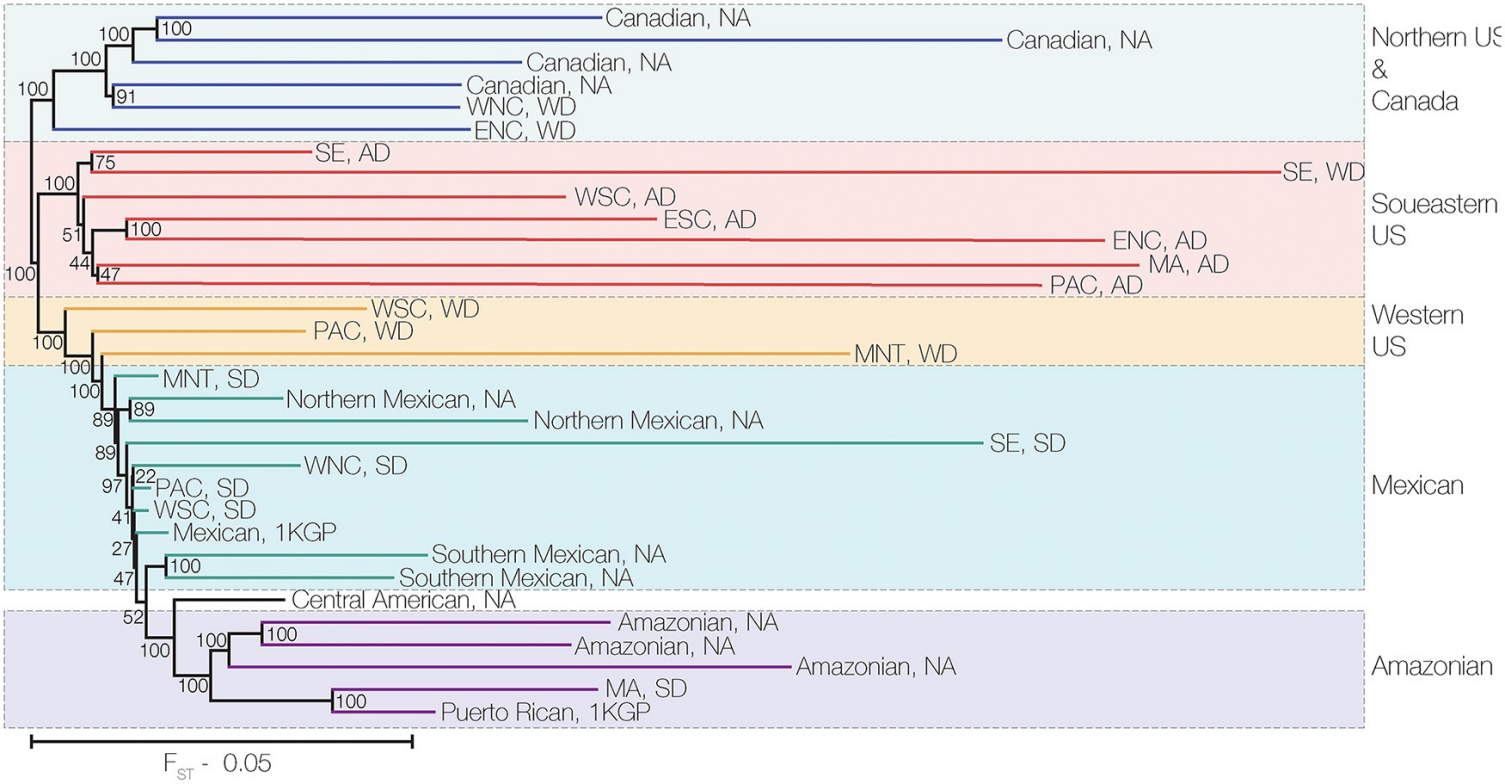
Native American ancestry phylogeny. Phylogenetic relationships are shown for the Native American ancestry-specific components of Native American (NA) reference populations, 1000 Genomes Project reference populations (Mexican and Puerto Rican) and HRS groups. The HRS groups are labeled according to their US census region origins and genetic ancestry group: African descendant (AD), Western European descendant (WD), and Spanish descendant (SD). Broad geographic and genetic groupings are indicated by the bars on the right side. The scale bar corresponds to the pairwise F_ST_ values used to generate the phylogeny.

The phylogenetic relationships of the Native American ancestry in modern US populations were inferred by calculating the fixation index (F_ST_) between pairs of populations based on their masked Native American haplotypes (Fig 5). The Canadian and Amazonian Native American reference populations occupy the most distant clades on the phylogeny with the admixed Mexican and Mexican Native American reference populations adjacent to the Amazonian group. African descendant populations from all of the census regions form a single clade, along with Western European descendants from the Southeast region (SE, WD). Western European descendant populations from the West North-Central (WNC, WD) and East North-Central (ENC, WD) regions group most closely with the Canadian Native American reference populations. Western European descendant populations from the Western US (West South-Central (WSC, WD), Pacific (PAC, WD), and Mountain (MNT, WD) regions) are intermediate between the African descendant clade and the Spanish descendant of populations. Spanish descendant populations from most of the US census regions group closely with Mexican populations, with the exception of the Mid-Atlantic region (MA) which groups most closely with the Puerto Rican and Amazonian reference populations. To quantify the affinities of the Native American ancestry in admixed US populations we computed outgroup f_3_-statistics of the form *f*_3_(African; admixed, reference) and D-statistics of the form *D*(African, admixed; Native American, reference) using the masked Native American haplotypes and AdmixTools [13]. The *f*_3_ and *D*-statistics agree well with the inferred phylogeny (S14 & S15 Figs). Western European descendants from WNC and ENC regions showed the highest affinity for Canadian Native American populations. Consistent with the single clade observed in the phylogeny, African descendant populations showed generally lower affinity to the reference populations. Spanish descendant populations showed the highest affinities for Mexican reference populations, apart from the MA population which showed higher affinity for Amazonian groups.

### Native American ancestry of the *Nuevomexicanos*

**Fig 6:**
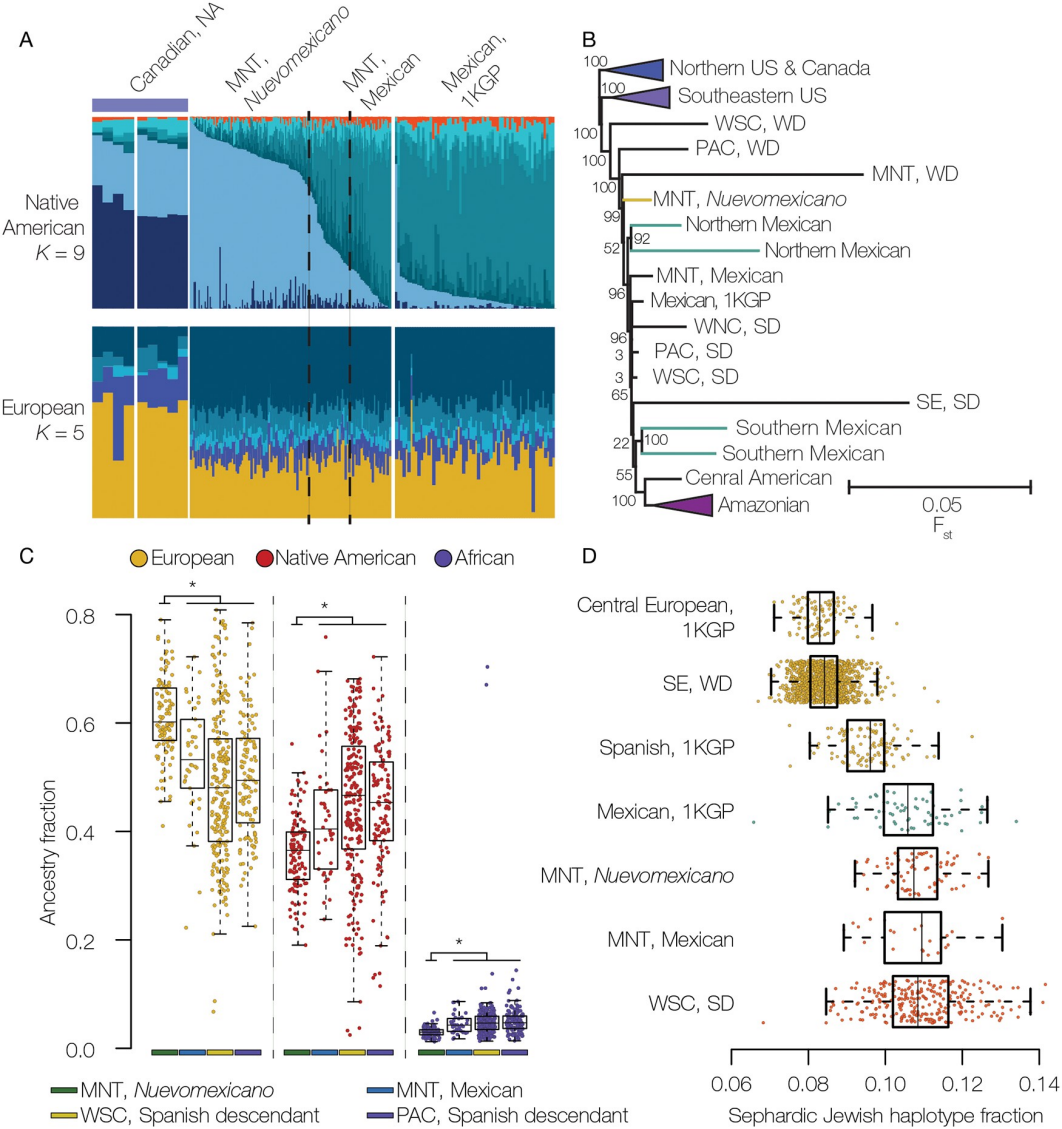
Genetic ancestry of the *Nuevomexicanos*. (A) Native American (*K* = 9) and European (*K* = 5) ancestry-specific ADMXITURE plots comparing the Mountain census region (MNT) in the middle panel to Canadian Native American (NA) populations and admixed Mexican reference populations. Native American ancestry profiles for the Mountain region can be divided into *Nuevomexicano* (left) and Mexican-American (right) components. (B) Native American ancestry phylogeny (as shown in Fig 5) with the Mountain census region (MNT) broken down into *Nuevomexicano* and Mexican-American sub-populations. (C) Distributions of European, Native American, and African ancestry fractions are shown for the Spanish descendant Mountain (MNT) *Nuevomexicano*, Mountain (MNT) Mexican, West South Central (WSC), and Pacific (PAC) populations. The * indicates significant differences in median ancestry fractions between the *Nuevomexicano* and other groups (*P*<0.01 Wilcoxon Rank-Sum test). (D) Distributions of the Sephardic Jewish haplotype copying fractions are shown for European reference populations from the 1000 Genomes Project (Central European and Spanish), Western European descendants from the Southeast census region (SE, WD), Mountain (MNT) *Nuevomexicano*, Mountain (MNT) Mexican, and West South Central Spanish descendant (WSC, SD) groups.

The ADMIXTURE results for the Spanish descendant group in the Mountain region (MNT) point to the presence of two distinct sub-populations, one of which is clearly of Mexican descent, whereas the second group has a pattern distinct from any other group analyzed here (Figs 4B and 6A). If these two apparent Spanish descendant Mountain sub-populations are considered separately, they form distinct phylogenetic groups (Fig 6B). One group clearly falls into the clade with the other Mexican origin populations (MNT, Mexican), whereas the distinct group is basal to the Mexican clade and intermediate between the Western US and Mexican clades (MNT, *Nuevomexicano*). The results of the ADMIXTURE and phylogenetic analyses are consistent with historical records indicating the presence of a unique group of Spanish descendants in the American Southwest, known as the ‘Hispanos of New Mexico’ or *Nuevomexicanos*. This population is descended from very early Spanish settlers to the Four Corners region of the US, primarily New Mexico and southern Colorado, and distinct from Mexican-American immigrants who arrived later [14].

Members of the *Nuevomexicano* population have maintained a distinct cultural identity for centuries, and the ability to isolate individuals from this group based on analysis of their genotypes allowed us to address open questions related to their ancestry. In addition to characterizing their distinct pattern of Native American ancestry, we also compared the levels of Native American admixture between *Nuevomexicanos* and the other nearby Spanish descendant groups, which show a Mexican pattern of Native American ancestry. Consistent with previous results [15], we show that *Nuevomexicanos* have significantly more European ancestry and less Native American ancestry than other Spanish descendant groups from the Western Census regions (Fig 6C). *Nuevomexicanos* also show significantly lower levels of African ancestry compared to the other Spanish descendant groups.

*Nuevomexicano* cultural and historical traditions suggest that many of the early Spanish settlers in the region were *Conversos*, Jewish individuals who ostensibly converted to Catholicism in an effort to avoid religious persecution and pogroms, while secretly maintaining Jewish identity and traditions [16]. We interrogated this idea by comparing the extent of Sephardic Jewish admixture found among individuals with the *Nuevomexicano* ancestry pattern compared to other Spanish descendant populations. Sephardic Jewish admixture was measured by comparing European haplotypes from Spanish descendant individuals to a reference panel including both European and Sephardic Jewish populations. *Nuevomexicanos* show elevated levels of matching to Jewish haplotypes compared to Spanish and other European populations, consistent with substantial *Converso* ancestry among New World Spanish descendant populations [17] (Fig 6d). However, *Nuevomexicanos* do not show a higher level of *Converso* ancestry compared to the other New World Spanish descendant populations.

## Discussion

### Native American admixture patterns for distinct US ancestry groups

We were able to delineate three predominant genetic US ancestry groups–African descendant, Western European descendant, and Spanish descendant–using comparative analysis of whole genome genotypes from >15,000 individuals from across the continental US. Each of these different groups of people experienced distinct historical trajectories in the US, which we found to be manifested as group-specific patterns of Native American ancestry.

Individuals from the African descendant group show low (Fig 1B) and relatively invariant (Fig 3A) levels of Native American ancestry across the continental US. The patterns of Native American ancestry seen for the African descendant group are also more constant among US census regions compared to individuals from the other two ancestry groups (Fig 4A). With respect to the Native American component of their ancestry, African descendant populations from all US census groups form a single clade, along with the Southeast Western European descendant population (SE, WD) (Fig 5). Considered together, these results point to a most likely scenario whereby African descendants admixed with local Native American groups in the antebellum South. Early admixture with Native Americans in the South was followed by subsequent dispersal across the US during the Great Migration in the early to mid-twentieth century [18]. The genetic legacy of the Great Migration has previously been explored based on overall patterns of African American genetic diversity [19]. Here, we were able to uncover traces of this same history based solely on the relatively low Native American ancestry component found in the genomes of African descendants.

Of the three US ancestry groups characterized here, the Western European descendant group shows the lowest levels of Native American ancestry (Fig 1B), consistent with a large and fairly constant influx of European immigrants to the US along with social and legal prohibitions against miscegenation [20]. Compared to African descendants, individuals from the Western European descendant group show more variant levels of Native American ancestry among US census regions (Fig 3B) along with substantially more region-specific patterns of Native American ancestry (Fig 4A). Their region-specific patterns of Native American ancestry are also reflected in the Native American ancestry-based phylogeny, whereby the Western European descendant populations are related according to their geographic origin across the country (Fig 5). These results point to a historical pattern of continuous, albeit infrequent, admixture between local Native American groups and European settlers as they moved westward across the continental US.

As can be expected, the Spanish descendant group shows by far the highest (Fig 1B) and most variable (Fig 3C) levels of Native American ancestry across the US. Individuals from this group show highly regional-specific patterns of Native American ancestry (Fig 4B), consistent with known demographic trends. For example, analysis of the Native American component of Spanish descendant ancestry is sufficient to distinguish Puerto Rican immigrants from the Mid-Atlantic census region from Mexican Americans who predominate in the western census regions. Perhaps most striking, the patterns of Native American ancestry seen for the Mountain census regions were alone sufficient to distinguish descendants of very early Spanish settlers to the region, the group known as Hispanos or *Nuevomexicanos*, from subsequent waves of Spanish descendants who arrived later from Mexico.

The three main US ancestry groups are also distinguished by their patterns of sex-biased ancestry in a way that reflects the unique history of each group (Fig 2). Western European descendants show very little evidence for sex-biased ancestry, along with very low levels of overall admixture, compared to the African and Spanish descendant groups. Sex-bias for Spanish descendants is characterized by a strong female-bias for Native American ancestry coupled with European male-biased ancestry. The pattern that we observe here is similar to what has been reported in a number of previous studies and is consistent with the history of male-biased migration to the region dating back to the era of the conquistadors [21, 22]. The African descendant group shows female-biased African ancestry and male-biased European ancestry, a pattern which has also been documented previously and tied to the legacy of slavery and racial oppression in the US [23, 24]. It has not been previously possible to directly compare the extent of sex-biased admixture among the three largest ancestry groups in the US as we have done here. As such, it is interesting to note that the history of the Spanish colonization in Latin America had a stronger impact on sex-biased ancestry than the legacy of slavery in the US.

### Implications of genetic ancestry for the historical and cultural traditions of *Nuevomexicanos*

Our ability to distinguish *Nuevomexicanos* from the HRS dataset, using their distinct Native American ancestry, allowed us to address a number of open questions and controversies regarding the history and culture of this interesting population. *Nuevomexicanos* from the American southwest are historically defined as the descendants of early Spanish settlers, those who arrived in the period from 1598 to 1848, as opposed to immigrants from Mexico who arrived the region considerably later. The two distinct patterns of Native American ancestry seen for Spanish descendant individuals from the Mountain census region are very much consistent with this historical definition. The *Nuevomexicanos* show a pattern of Native American ancestry that is intermediate to the Canadian and Mesoamerican reference populations analyzed here, whereas the Mexican American individuals from the same region are more closely related to Mesoamerican reference populations. This is consistent with early admixture with local Native American groups in the US southwest, for the *Nuevomexicanos*, versus admixture with Mesoamerican groups in Mexico for the later Mexican immigrants. A more precise characterization of *Nuevomexicanos’* Native American ancestry would require access to genomic data from US Native American reference populations, which are not readily available owing to cultural resistance to genetic testing for ancestry among these groups [25].

Historically, *Nuevomexicanos* have identified strongly with their European (Spanish) ancestry, while downplaying ancestral ties to Native Americans [26]. This tradition of exclusive European identity is rooted in the colonial era when Spanish descendants in the region were preoccupied with the notion of maintaining so-called pure blood, and the local aristocracy identified as Castilian. The Spanish preoccupation with admixture in the Americas was codified into the so-called *Sistema de Castas*, whereby mixed-race individuals were categorized into a complex hierarchical system, with tangible legal and social implications, based on their parents’ ancestry [27]. Mexicans, on the other hand, have long identified as *Mestizo* with an explicit recognition of their Native American heritage [28]. Our comparative analysis of genetic ancestry for *Nuevomexicanos* and Mexican ancestry groups yielded results that are partly consistent with this historical narrative. On the one hand, *Nuevomexicanos* do have a substantial amount of Native American ancestry, with a median of just under 40% (Fig 6C), which is far more than seen for the African descendant and Wester European descendant groups analyzed here. The fraction of Native American ancestry seen in the *Nuevomexicanos* is also higher than in several populations in South America (Medellín [29] and Chocó [30], Colombia) and the Caribbean (Cuba, the Dominican Republic, and Puerto Rico [29]). Nevertheless, the *Nuevomexicanos* have significantly less Native American ancestry, and more European ancestry, than nearby Mexican descendant populations (Fig 6C). Our results are consistent with a recent study that used microsatellite-based ancestry analysis on a much smaller sample of self-identified *Nuevomexicanos*, who were also found to have higher European ancestry and lower Native American ancestry compared to Mexican Americans [15]. Interestingly, we found that the *Nuevomexicanos* also have significantly less African ancestry than Mexican descendant populations, which likely reflects higher levels of early African admixture in Mexico [31].

We investigated this apparent differing population history by inferring the timings and proportions of admixture with the TRACTS utility [32]. The best models from the TRACTS analysis indicated a European and Native American admixture 10–11 generations ago, followed shortly by a small African admixture (S16 Fig). All models for the Mexican populations converged on an admixture time of 10–11 generations. The best *Nuevomexicano* model suggests a slightly older admixture, though with the same ordering, 11–12 generations ago, while the best *Nuevomexicano* model for 10–11 generations produced a significantly worse model (log-likelihood of -413 vs. -390). Regardless, this suggests that the timing of admixture in the Mexican populations and the *Nuevomexicano* population was similar, consistent with historical records, while the Native American source populations were different, consistent with their geographical origins. While the admixture timing estimates for these groups are within the range of previous estimates, they are younger than what has been previously reported for Mexican populations [33]. Nevertheless, as can be expected for a Caribbean population, the Puerto Rican descendant MA population showed a much older admixture, ~15 generations ago, very similar to the 1KGP Puerto Rican population (S17 Fig).

Perhaps the most controversial aspect of *Nuevomexicano* history relates to the influence of *Conversos* on the culture and traditions of the local community. *Conversos* are Jewish people who converted to Catholicism under intense pressure from religious persecution in Spain, and elsewhere in Europe, and many Spanish *Conversos* immigrated to the New World [34]. Despite their forced conversion to Catholicism, some New World *Conversos* apparently maintained Jewish religious traditions over the centuries since their immigration from Spain. For example, the persistence of rituals and symbols related to Jewish traditions in New Mexico has been taken as evidence for an influential presence of *Conversos* among the *Nuevomexicanos*, a position championed by the historian Stanley Hordes[16]. On the other hand, the folklorist Judith Neulander and others have been fiercely critical of this narrative based on what they perceive to be misunderstandings of the origins of many of the cultural traditions tied to Jewish rituals and even deliberate misrepresentations of facts [35]. Neulander’s interpretation relates the notion of *Converso* identity among *Nuevomexicanos* back to the colonial assertions of pure Spanish ancestry given that the Sephardim are Spanish and would presumably be loath to marry outside of their religious group [36].

We evaluated the extent of Sephardic Jewish ancestry among *Nuevomexicanos*, via comparative analysis of their European haplotypes to both European and Sephardic Jewish reference populations, in attempt to assess the genetic evidence in support of the *Converso* narrative. While we did find more Sephardic Jewish ancestry among *Nuevomexicanos* compared to Spaniards or other Europeans, they did not show any more Sephardic Jewish ancestry than Mexican descendants from nearby regions (Fig 6D). Our results are consistent with a recent study that used haplotype-based ancestry methods to uncover widespread *Converso* ancestry in Latin American populations [17]. Taken together, we interpret these results to indicate that, while *Nuevomexicanos* do in fact have a demonstrable amount of Jewish ancestry, they show no more, or less, Jewish ancestry than other New World Latin American populations. Of course, we cannot weigh in on the strength of evidence for or against the persistence of Jewish cultural traditions among *Nuevomexicanos* based on our genetic evidence alone. Nevertheless, there does not seem to be anything particularly unusual, at least from the genetic perspective, with respect to the extent of Sephardic Jewish heritage among *Nuevomexicanos*.

### Conclusion

Much of the genetic legacy of the original inhabitants of the area that is now the continental US can be found in the genomes of the descendants of European and African immigrants to the region. In this study, we analyzed signals of Native American genetic ancestry in a comparative analysis of genomes from the three largest US ancestry groups: African descendants, Western European descendants, and Spanish descendants. Our study was enabled by the use of haplotype-based methods for genetic ancestry inference and leveraged a large dataset of whole genome genotypes. This approach allowed for detailed analysis of Native American ancestry patterns even when the per-genome levels of Native American ancestry were quite low. Each of the three genetic ancestry groups analyzed here shows distinct profiles of Native American ancestry, which reflect population-specific historical patterns of migration and settlement across the US. Analysis of the Native American ancestry component for members of these groups allowed for the delineation of region-specific subpopulations, such as the *Nuevomexicanos* from the American southwest, and facilitated the interrogation of specific historical scenarios.

## Materials and methods

### Ethics statement

This study was approved by the Georgia Institute of Technology Central Institutional Review Board, #H17029. Data were provided by third party sources and no additional ethical approval was required.

### Genotype datasets

Whole genome genotype data of US individuals from the Health and Retirement Study (HRS) dataset (*n* = 15,620) were merged with whole genome sequence variant data from the 1000 Genomes Project (1KGP) [37, 38] (*n* = 1,718) and whole genome genotype data from the Human Genome Diversity Project (HGDP) [12, 39, 40] (*n* = 230) (S1 Table). Individual HRS genotypes are provided along with geographical origin data for sample donors from the nine census regions in the continental US. A collection of Native American genotypes from 21 populations across the Americas was taken from a comprehensive study on Native American population history [2] (*n* = 314). These Native American genotype data were accessed according to the terms of a data use agreement from the Universidad de Antioquia. Whole genome genotype data from 5 populations of Sephardic Jewish individuals (*n* = 40) were also included as reference populations [41]. The genotypes from HRS individuals were merged with the comparative genomic data sources using PLINK version 1.9 [42], keeping only those sites common to all datasets and correcting SNP strand orientations for consistency as needed. The final merged dataset includes 228,190 SNPs across 17,882 individuals. Pairwise distances between individuals was calculated using the–dist option of PLINK [42], and principal component analysis carried out using the prcomp function of R [43]. The merged genotype dataset was phased using ShapeIT version 2.r837 [44]. SNPs that interfered with the ShapeIT phasing process were excluded from subsequent analyses. ShapeIT was run without reference haplotypes, and all individuals were phased at the same time. Individual chromosomes were phased separately, and the X chromosome was phased with the additional ‘-X’ flag.

### Local ancestry inference

The RFMix algorithm [45] is able to accurately characterize the local ancestry of admixed individuals but is prohibitively slow when run on a dataset of the size used here. To reduce the runtime, we modified RFMix version 1.5.4 so that the expectation-maximization (EM) procedure samples from, and creates a forest for, the entire set of individuals rather than each individual. This modified RFMix was run in the PopPhased mode with a minimum node size of five, using 12 generations and the “—use-reference-panels-in-EM” for two rounds of EM, generating local ancestry inference for both the reference and admixed populations. Continental African, European, and Native American populations were used as reference populations. Contiguous regions of ancestral assignment, “ancestry tracts,” were created where RFMix ancestral certainty was at least 95%. Genome-wide ancestry estimates from the modified RFMix algorithm closely correlate with those from ADMIXTURE (S18 Fig). The present Native American reference populations may not be close to the actual ancestral Native American populations for all of the HRS regions. To evaluate how a distant reference population would affect the LAI, we carried out the RFMix procedure a second time, but using only East Asian populations as the reference for Native American ancestry. The local ancestry inferred in this was very similar to that inferred when using actual Native American populations as references (S19 Fig), indicating that the choice of reference population does not greatly affect the LAI.

### Haplotype heterozygosity

For each admixed population, rephased genotypes from the final output of RFMix were used to compute the haplotype heterozygosity (HH) for both the masked ancestry-specific genomes and for the unmasked whole-genome. Haplotypes were found by considering sets of 5–15 consecutive variants with a maximum recombination rate between any two variants of 0.5 cM/mB as in [46], resulting in 11,816 haplotypes. Significance in HH between ancestry-specific genomes was assessed using a Wilcoxon rank-sum test.

### Sephardic Jewish ancestry

The extent of Sephardic Jewish (*Converso*) ancestry in individuals from the Spanish descendant group in HRS (as defined in the genome-wide ancestry section below), and Latin American populations from 1KGP, was inferred via ancestry-specific haplotype comparisons with Sephardic Jewish reference populations using the program ChromoPainter2 [10] (kindly provided by Garrett Hellenthal). First, African and Native American haplotypes were masked from the RFMix output. Then, the remaining European haplotypes were compared against genomes from the European reference populations together with the Sephardic Jewish populations. The extent of Jewish ancestry for any individual genome is defined as the ‘copying fraction’ from the Sephardic Jewish populations, where the copying fraction is taken as the fraction of sites with best matches to the Sephardic Jewish reference genomes. It should be noted that this procedure results in a relative fraction of Sephardic Jewish ancestry for all individuals under consideration, which is directly comparable among individuals but likely to be an overestimate of the total ancestry derived from a single source population.

### Genome-wide ancestry inference

ADMIXTURE [47] version 1.3.0 was used with *K* = 4 to infer continental ancestry fractions for individuals in the dataset via comparison with reference populations from Africa, Europe, the Americas, and East Asia. Sub-continental ancestry was inferred independently for each of the three major continental ancestry components–African, European, and Native American–using an ancestry-specific masking procedure that we developed as previously described [30]. This procedure relies on the local continental ancestry assignments, along with the re-phased genotypes, generated by RFMix as described above. Sub-continental ancestry was characterized by first masking out two of the three continental ancestries (African, European, and/or Native American) at a time and then analyzing the genomic regions (haplotypes) corresponding to the remaining continental ancestry. For sub-continental ancestry analysis of any given continental ancestry component, only those individuals with at least 1.5% genome-wide ancestry for that same continental group were used. This 1.5% threshold was chosen empirically based on observed ancestry assignments in the reference populations. As this work was focused on Native American ancestry, we chose a threshold higher than the Native American ancestry inferred in any of the European or African reference populations (max = 1.4% in a Spanish individual). While lowering this threshold would likely include a number of additional individuals with genuine Native American ancestry, we chose this stricter cutoff to avoid any possible ambiguity.

We developed a novel machine learning based approach to distinguish Spanish from other (primarily Western) European descendants in the HRS dataset via analysis of European-specific haplotypes. First, ADMIXTURE was run with *K* = 5 on the RFMix characterized European haplotypes for the HRS individuals to stratify sub-continental European ancestries based on comparison with Northern (Finnish and Russian), Western (French and British), Spanish, and Southern (Italian and Sardinian) European reference populations from the 1KGP and HGDP datasets. The ADMIXTURE results at *K* = 5 were used as one of the ADMIXTURE components was substantially different between the Spanish and Italian reference populations (Fig 4, S3 and S4 Figs). A Support Vector Machine (SVM) classifier [48] was then trained using the resulting ADMIXTURE ancestry vectors for the European reference populations from the four sub-continental groups: Northern, Western, Spanish, and Southern. The European-specific ADMIXTURE ancestry vectors for the HRS individuals were then classified into one of the four European sub-continental groups defined by the SVM classifier. A confidence threshold of 0.8 was used for sub-continental group assignments in order to minimize the number of misclassified individuals; while a lower threshold would allow for additional individuals to be included, a threshold below 0.7 lead to a higher missassignment rate while validating the classifier. For the purpose of analysis here, we consider two major groups of European descendants in the HRS data set: Spanish descendants (SD) and all others. Non-Spanish HRS individuals with <5% African ancestry are defined as Western European descendant (WD), whereas non-Spanish HRS individuals with at least 20% African ancestry were defined as African descendant (AD). It should be noted that this approach to defining genetic ancestry groups, as opposed to relying on self-identified race/ethnicity groups, is likely to yield ancestry classifications that correspond very well to self-identified race/ethnicity labels for the vast majority of individuals analyzed here. But our African descendant group will not include a small fraction of self-identified African Americans with little or no African ancestry. For example, there are 11 individuals in HRS who self-identify as African American but have no discernable African ancestry. We chose to rely on genetic ancestry, as opposed to self-identified race/ethnicity, in such cases in an effort to be as consistent as possible when delineating the three broad ancestry groups. We discuss this issue at more length in the Supplementary material (S1 Text).

### Sex-biased ancestry inference

Sex-biased ancestry contributions were inferred by comparing the RFMix characterized fractions of each continental ancestry component on the X chromosomes versus the autosomes as previously described [22, 46]. For each individual genome, and each ancestry component, the normalized difference between the X chromosome ancestry fraction and the autosomal ancestry fraction (Δ*Admix*) is defined as:

$$\Delta Admix=F_{anc,total}\times (F_{anc,X}-F_{anc,auto})/(F_{anc,X}+F_{anc,auto})$$

where *F*_*anc*,*total*_, *F*_*anc*,*X*_, and *F*_*anc*,*auto*_ are the genome-wide, X chromosome, and autosome ancestry fractions, respectively.

### Phylogenetic inference

We used the RFMix defined Native American haplotypes for individuals from the HRS and reference populations to infer the phylogenetic relationships between populations. Using the masked Native American haplotypes, the F_ST_ was found between each population using smartpca from the EIGENSOFT package [49]. The resulting F_ST_ distance matrix was used to create a neighbor-joining tree [50] with the program MEGA6 [51]. Clade bootstrap values were calculated by resampling sites from the data, recalculating F_ST_, and counting the occurrences of each clade using prop.part and part.clades of the Ape package [52].

### Admixture timing

The TRACTS method was used to infer the timing of admixture events with ancestry tracts defined by RFMix [32]. For the admixed *Nuevomexicano*, Mexican (1KGP), MA Spanish descendant, and Puerto Rican (1KGP) populations, three possible orderings of admixture were evaluated with TRACTS: (1) European, Native American, and African; (2) European, African, and Native American; and (3) African, Native American, and European. For each ordering, TRACTS was used to evaluate possible admixture timing from 14 to six generations ago, in 1000 bootstrap attempts. From the bootstrap attempts, the most likely series of admixture events was chosen to represent the population.

## Supporting information

S1 Text Supporting information and methods. The methods used to assign genetic ancestry groups to HRS individuals and the CLUMPP-ADMIXTURE analysis.
(DOCX)

S1 Table Populations and sources used in this analysis. Populations included and the number of individuals in each population are shown. How or if a population was used for local or sub-continental ancestry analysis is indicated.
(DOCX)

S2 Table Cross-validation of the European ancestry classifier. A support vector machine (SVM) was created to characterize the European ancestry of individuals using ADMIXTURE values generated using masked European genotypes. 10-fold cross-validation was used to evaluate the performance of the SVM. Values shown are the numbers of individuals assigned to each ancestry in the validation procedure. Correct assignments are on the diagonal.
(DOCX)

S3 Table Validation of the European ancestry classifier on New World populations. The SVM was used to characterize New World individuals with known European ancestry. Values shown are the number of individuals assigned each European ancestry from each population. For each population, the known European ancestry is listed in the parentheses.
(DOCX)

S4 Table Haplotype heterozygosities of ancestry-specific and whole-genome haplotypes. Haplotype heterozygosities (HH) were found for both the ancestry-specific and genome and whole-genome for each of the admixed populations.
(DOCX)

S1 Fig Map showing the nine US census regions used to assign the geographic origins of the HRS individuals analyzed here. The census regions, in semi-clockwise order, are: West North-Central (WNC), East North-Central (ENC), Northeast (NE), Mid-Atlantic (MA), Southeast (SE), East South-Central (ESC), West South-Central (WSC), Mountain (MNT), and Pacific (PAC).
(TIF)

S2 Fig ADMIXTURE plot showing HRS individuals’ continental ancestry fractions. African (blue), European (yellow), Native American (red), and East Asian (green) ancestry components are shown. The HRS individuals were divided into two groups based on their self-identified status as African-Americans and all others. HRS individuals shown in comparison to African (African American–ASW), European (Central European–CEU), Latin American (Mexican–MXL and Puerto Rican–PUR) 1KGP reference populations.
(TIF)

S3 Fig ADMIXTURE analysis of European haplotypes for HRS individuals classified by genetic ancestry as African descendant (AD) or Western European descendant (WD). Individuals were placed into genetic ancestry groups using the SVM classifier as described in the Materials and Methods. ADMIXTURE was run and HRS individuals from the two genetic ancestry groups (AD-purple and WD-lime) are plotted according to their geographic region of origin along with individuals from European (Finnish, British, French, Spanish, and Italian) and Mexican 1KGP reference populations. ADMIXTURE was run using *k* = 2, 3, 4, and 5 populations.
(TIF)

S4 Fig ADMIXTURE analysis of European haplotypes for HRS individuals classified by genetic ancestry as Spanish descendant (SD). Individuals were placed into genetic ancestry groups using the SVM classifier as described in the Materials and Methods. ADMIXTURE was run and HRS individuals from the Spanish descendant group are plotted according to their geographic region of origin along with individuals from European (Finnish, British, French, Spanish, and Italian), Mexican, and Puerto Rican 1KGP reference populations. ADMIXTURE was run using k = 2, 3, 4, and 5 populations.
(TIF)

S5 Fig CLUMPP analysis of European ADMIXTURE from Western European descendant (WD) and African descendant (WD) populations. ADMIXTURE was run on masked European haplotypes from WD and AD individuals 20 times for *K* = 2 to *K* = 5, using different seeds for each run. The CLUMPP utility was used to identify corresponding inferred ancestries across ADMIXTURE runs that used the same *K*. The means of the CLUMPP characterized ancestries are shown here.
(TIF)

S6 Fig CLUMPP analysis of European ADMIXTURE from Spanish descendant (SD) populations. ADMIXTURE was run on masked European haplotypes from SD individuals 20 times for *K* = 2 to *K* = 5, using different seeds for each run. The CLUMPP utility was used to identify corresponding inferred ancestries across ADMIXTURE runs that used the same *K*. The means of the CLUMPP characterized ancestries are shown here.
(TIF)

S7 Fig Similarity in European ADMIXTURE vectors between populations. For each population, the mean CLUMPP-ADMIXTURE vector was found by concatenating the vectors from *K = 2* to *K* = 5 and taking the mean of each component across individuals. The Euclidean distance in these vectors was found between all populations, and rescaled from 0 to 1. The similarity between populations was found as 1—the distance.
(TIF)

S8 Fig Comparison of European ADMIXTURE similarity between US populations. European CLUMPP-ADMIXTURE similarities were found between African descendant (AD), Western European descendant (WD), and Spanish descendant (SD) populations. Differences in similarity between two population groups were assessed using a Wilcoxon rank-sum test.
(TIF)

S9 Fig Comparison of haplotype heterozygosity between ancestry-specific and whole-genome haplotypes. Haplotype heterozygosity (HH) values were found for the ancestry-specific genomes and whole-genomes of admixed populations. For each of the admixed populations, the HH value for each of the three continental ancestries were compared to the whole-genome HH value. Significance was assessed using a Wilcoxon rank-sum test.
(TIF)

S10 Fig CLUMPP analysis of Native American ADMIXTURE from Western European descendant (WD) and African descendant (AD) populations. ADMIXTURE was run on masked Native American haplotypes from AD and WD individuals 20 times for *K* = 2 to *K* = 9, using different seeds for each run. The CLUMPP utility was used to identify corresponding inferred ancestries across ADMIXTURE runs that used the same *K*. The means of the CLUMPP characterized ancestries are shown here.
(TIF)

S11 Fig CLUMPP analysis of Native American ADMIXTURE from Spanish descendant (SD) populations. ADMIXTURE was run on masked Native American haplotypes from HL individuals 20 times for *K* = 2 to *K* = 9, using different seeds for each run. The CLUMPP utility was used to identify corresponding inferred ancestries across ADMIXTURE runs that used the same *K*. The means of the CLUMPP characterized ancestries are shown here.
(TIF)

S12 Fig Similarity in Native American ADMIXTURE vectors between populations. For each population, the mean CLUMPP-ADMIXTURE vector was found by concatenating the vectors from *K = 2* to K = 9 and taking the mean of each component across individuals. The Euclidean distance in these vectors was found between all populations, and rescaled from 0 to 1. The similarity between populations was found as 1—the distance.
(TIF)

S13 Fig Comparison of Native American ADMIXTURE similarity between US populations. Native American CLUMPP-ADMIXTURE similarities were found between African descendant (AD), Western European descendant (WD), and Spanish descendant (SD) populations. Differences in similarity between two population groups were assessed using a Wilcoxon rank-sum test.
(TIF)

S14 Fig Comparison of admixed US populations to reference Native American populations by the *f*_3_ statistic. Masked Native American genotypes were used to compare admixed US populations to Native American reference populations using the outgroup *f*_*3*_ statistic. The *f*_3_-statistics were computed as *f*_3_(YRI; admixed, reference).
(TIF)

S15 Fig Comparison of admixed US populations to reference Native American populations by the *D* statistic. Masked Native American genotypes were used to compare admixed US populations to Native American reference populations using the *D* statistic. The *D*-statistics were computed as *D*(YRI, admixed; Pima, reference) to determine whether admixed populations were more closely related to the modern Pima population or to another Native American population.
(TIF)

S16 Fig Admixture timing using tracts analysis in the *Nuevomexicano* SD population and Mexican population. (Left side) Observed (points) and predicted (solid line) ancestry tract size distributions; the shaded areas represent 95% confidence intervals. (Right side) Admixture event timings are shown together with ancestry proportions. Each inferred admixture event is indicated by a circle, which is scaled according to the size of the contribution to the population and also shows the relative ancestry proportions. The y-axes of the charts show the inferred continental ancestry fractions, and the x-axes show time as the number of generations ago (GA). (A) Inferred admixture timing in the MNT *Nuevomexicano* population. (B) Inferred admixture timing in the 1KGP Mexican population.
(TIF)

S17 Fig Admixture timing using tracts analysis in the MA SD population and Puerto Rican population. (Left side) Observed (points) and predicted (solid line) ancestry tract size distributions; the shaded areas represent 95% confidence intervals. (Right side) Admixture event timings are shown together with ancestry proportions. Each inferred admixture event is indicated by a circle, which is scaled according to the size of the contribution to the population and also shows the relative ancestry proportions. The y-axes of the charts show the inferred continental ancestry fractions, and the x-axes show time as the number of generations ago (GA). (A) Inferred admixture timing in the Mid-Atlantic Spanish descendant population. (B) Inferred admixture timing in the 1KGP Puerto Rican population.
(TIF)

S18 Fig Comparison of the modified RFMix to ADMIXTURE for inferring continental ancestry. The RFMix utility was modified to reduce the computational time. The resulting genome-wide continental ancestry fractions (as determined by summing the local ancestry across each individual) were compared to estimates given by ADMIXTURE for (A) African ancestry, (B) European ancestry, and (C) Native American ancestry. All correlations between RFMix and ADMIXTURE continental ancestry fractions were significant (*p < 1e-10*, Pearson linear correlation).
(TIF)

S19 Fig Continental ancestry inferred using an only East Asian reference populations as surrogate for Native American populations. The modified RFMix utility was used to infer local ancestry as above in S18 Fig, however, modern East Asian populations were used as the reference for Native American ancestry rather than Native American populations. The genome-wide continental ancestry fractions were compared between this East Asian reference population analysis and those generated using Native American reference populations for (A) African ancestry, (B) European ancestry, and (C) Native American ancestry. All correlations of continental ancestry between the two RFMix analyses fractions were significant (*p < 1e-10*, Pearson linear correlation).
(TIF)
