# Supplementary material for: Native American admixture recapitulates population-specific migration and settlement of the continental United States
Source: PLoS Genet. 2019 Sep 23;15(9):e1008225. doi: 10.1371/journal.pgen.1008225 (PMC6756731; doi:10.1371/journal.pgen.1008225)
Supplement: S1 Text — The methods used to assign genetic ancestry groups to HRS individuals and the CLUMPP-ADMIXTURE analysis. (DOCX) [file pgen.1008225.s001.docx]

Supporting information for:

**Native American admixture recapitulates population-specific migration and settlement of the continental United States**

I. King Jordan, Lavanya Rishishwar, and Andrew B. Conley

**S1 Text.**

**Delineation of genetic ancestry groups in the US.** We compared whole genome genotypes from 15,620 HRS individuals to genotype data from African, European, Native American, and East Asian global reference populations in an effort to define major genetic ancestry groups. The individual HRS donors who contributed genotype data were placed into two broad groups by the study according to their self-identified race/ethnicity: African Americans and all others. We initially ran ADMIXTURE, comparing these two HRS-defined groups to global reference populations, in order to visualize the continental ancestry fractions – African, European, Native American, and East Asian – for individuals in each group (S2 Fig).

Self-identified African Americans from the HRS show a wide range of African ancestry (0 – 100%) with an average African ancestry of 81.7%. Interestingly, this group includes a number of individuals with anomalously low levels African genetic ancestry, including 11 individuals with little to no discernable African ancestry. The second HRS-defined group, containing all of the remaining genotype donors, shows a mix of individuals with primarily European ancestry along with a second large group that contains individuals with a mix of primarily European and Native American ancestry. That second group, which most likely corresponds to Hispanic/Latino individuals, also contains lower levels of African ancestry.

We wanted to classify HRS individuals from these two broad groups based on their observable patterns of genetic ancestry, as opposed to using self-identified race/ethnicity, which can be an unreliable indicator of ancestry. However, we did also consider previous studies, which have shown different overall levels of admixture for genetic ancestry groups in the US, when selecting criteria for genetic ancestry-based group definitions. For example, African-Americans can have substantial levels of European admixture, with an average of ~75-80% African ancestry and a long tail of increasing European ancestry, whereas European-Americans have very low levels of overall admixture and African ancestry in particular [1, 2]. Hispanic/Latino individuals tend to be more admixed with three-way ancestry contributions from Spain, the Americas, and Africa.

Individuals were characterized as having Western European ancestry or Spanish ancestry groups based on their sub-continental European ancestry. To do so, we considered only the European haplotypes for these individuals and compared them to haplotypes from (1) Spanish and (2) Finnish, British, French, and Italian European reference populations. As described in the Materials and Methods section, a support vector machine (SVM) classifier was trained on ADMIXUTRE vectors for these two groups of European populations and then the SVM was used to classify the HRS individuals as having primarily Spanish-European ancestry or other European ancestry. This was done for HRS individuals grouped by their geographic origins among nine census regions in the continental US (S3 Fig)

We evaluated the performance of the SVM by 10-fold cross-validation, using the 1KGP and HGDP populations with European ancestry: GBR, French, and Orcadian (Western), FIN and Russian (Northern), TSI and Sardinian (Southern), IBS, MXL and PUR (Spanish). In the cross-validation, we saw that 96.7% of individuals were correctly assigned to their subcontinental ancestry groups, 2.7% were unassigned, and 0.6% were misassigned (Table S2). Of those that were assigned (*i.e.*, not including those not assigned to a European group), 99.4% were assigned correctly. We further validated the classifier on New World 1KGP populations: CEU (Western), ASW (Western), CLM (Spanish), and PEL (Spanish). Here we saw that 94.2% of individuals were correctly assigned, 4.6% were unassigned, and 1.2% were misassigned (Table S3). Again, when excluding unassigned individuals, 98.7% of individuals were correctly assigned to their European ancestry.

Individuals classified by the ADMIXTURE-based SVM as having Spanish-European ancestry were considered as members of the Spanish descendant (SD) ancestry group, whereas individuals with other European ancestry are considered as belonging to the Western European descendant ancestry group. The ADMXITURE ancestry fractions (vectors) used for this classification can be seen in S4 Fig (Wester European descendant) and S5 Fig (Spanish descendant). Western European descendant (WD) individuals show European ancestry patterns that are similar to the British and French European reference populations, whereas the Spanish descendant individuals show European ancestry patterns that more closely resemble both the Spanish European reference population and the Mexican reference population. The mean F_ST_ between the inferred Western European descendant populations and Western European reference populations was 0.0002, versus 0.0028 when compared to the Spanish reference populations (*P* < 1e-10, Wilcoxon rank-sum), showing that the Western European descendant individuals have European ancestry significantly closer to Western European populations. Conversely, Spanish descendant populations showed a higher F_ST_ when compared to Western European reference populations than to Spanish populations (0.0021 vs 0.00021, *P <* 1e-10).

Western European HRS individuals were sorted into the African descendant (AD) ancestry group based on their observed African genetic ancestry; this group includes all individuals that have >20% African Ancestry and non-Spanish European ancestry. Individuals who had non-Spanish European ancestry and showed <5% African ancestry, including a small number of self-identified African Americans, were retained in the Western European descendant group. Individuals from the African descendant genetic ancestry group show European ancestry patterns that are similar to those seen for the Western European descendant groups and distinct to those of the Spanish descendant groups. Mean F_FS_ between African descendant populations and Western European reference populations was 0.0002 versus 0.0017 when compared to the Spanish reference populations (*P* < 1e-10), indicating that the European ancestry of the inferred African descendant populations is significantly closer to Western European populations.

**CLUMPP analysis of ADMIXTURE results**. Multiple ADMIXTURE runs of the same *K* can be characterized together using the CLUMPP[3] utility which identifies similar ancestral components across the runs. We used CLUMPP with the ADMIXTURE results for European ancestry (*K* = 2 to *K* = 5) and Native American ancestry (*K* = 2 to *K* = 9) (S5-S6 Fig and S10-S11 Fig, respectively). For each K, ADMIXTURE was run 20 times with a different seed used for each run. CLUMPP was then used to find the best matching ancestral components across the 20 different runs for each K, and to find the mean of those components across the runs. For each *K* used, the output of the CLUMPP-ADMIXTURE analysis produced a new vector of length *K* for each individual, representing the mean of each of the *K* components across the runs. CLUMPP-ADMIXTURE vectors from each of the different *K’s* were concatenated for each individual, and the values averaged across the individuals within each population. Euclidean distances were then found between all of these population-mean CLUMPP-ADMIXTURE vectors. Between-population distances were scaled to the range 0-1, and the similarity between populations found as 1 less the scaled distance (S7 Fig & S12 Fig). In the characterization of European ancestry (S7 Fig), African descendant and Western European descendant populations showed strong similarity to each other and to British and French reference populations. Conversely, Spanish descendant populations show stronger similarity to the Spanish reference population. The characterization of the Native American ancestry showed again that the African descendant and Western European descendant populations were similar to each other, though not as close as their European ancestry (S12 Fig). Spanish descendant populations were again distinct from the Western European descendant and African descendant populations and more similar to the Spanish and Mexican reference populations. Significant difference in similarity between African descendant and Western European descendant populations, African descendant and Spanish descendant populations, and Western European descendant population and Spanish descendant populations were assessed using a Wilcoxon rank-sum test (S8 Fig & S13 Fig). This testing showed that, under CLUMPP-ADMIXTURE analysis, African descendant and Western European descendant populations were generally more similar to each other under analysis than either was to Spanish descendant populations for both European ancestry (S8 Fig) and Native American ancestry (S13 Fig).

1. Kosoy R, Nassir R, Tian C, White PA, Butler LM, Silva G, et al. Ancestry informative marker sets for determining continental origin and admixture proportions in common populations in America. Hum Mutat. 2009;30(1):69-78. Epub 2008/08/08. doi: 10.1002/humu.20822. PubMed PMID: 18683858; PubMed Central PMCID: PMCPMC3073397.

2. Bryc K, Durand EY, Macpherson JM, Reich D, Mountain JL. The genetic ancestry of African Americans, Latinos, and European Americans across the United States. Am J Hum Genet. 2015;96(1):37-53. Epub 2014/12/23. doi: 10.1016/j.ajhg.2014.11.010. PubMed PMID: 25529636; PubMed Central PMCID: PMCPMC4289685.

3. Jakobsson M, Rosenberg NA. CLUMPP: a cluster matching and permutation program for dealing with label switching and multimodality in analysis of population structure. Bioinformatics. 2007;23(14):1801-6. Epub 2007/05/09. doi: 10.1093/bioinformatics/btm233. PubMed PMID: 17485429.
