# Supplementary material for: Native American admixture recapitulates population-specific migration and settlement of the continental United States
Source: PLoS Genet. 2019 Sep 23;15(9):e1008225. doi: 10.1371/journal.pgen.1008225 (PMC6756731; doi:10.1371/journal.pgen.1008225)
Supplement: S1 Table — Populations included and the number of individuals in each population are shown. How or if a population was used for local or sub-continental ancestry analysis is indicated. (DOCX) [file pgen.1008225.s002.docx]

| Population | N | Continental ancestry | Geographic group | Source |
| --- | --- | --- | --- | --- |
| African Caribbean | 94 | Admixed | Not used | 1000 Genomes |
| Aleutian | 8 | Native American | Not used | Reich et. al |
| Algonquin | 5 | Native American | Canadian | Reich et. al |
| Arhuaco | 5 | Native American | Not used | Reich et. al |
| African Ancestry in SW | 51 | Admixed | Not used | 1000 Genomes |
| Aymara | 23 | Native American | Not used | Reich et. al |
| Bantu, NE | 11 | African | Not used | HGDP |
| Bantu, SE, Pedi | 1 | African | Not used | HGDP |
| Bantu, SE, S.Sotho | 1 | African | Not used | HGDP |
| Bantu, SE, Tswana | 2 | African | Not used | HGDP |
| Bantu, SE, Zulu | 1 | African | Not used | HGDP |
| Bantu, SW, Herero | 2 | African | Not used | HGDP |
| Bantu, SW, Ovambo | 1 | African | Not used | HGDP |
| Cabecar | 31 | Native American | Not used | Reich et. al |
| CEU | 99 | European | Central European | 1000 Genomes |
| Han Chinese | 103 | Asian | Not used | 1000 Genomes |
| Chilote | 8 | Native American | Not used | Reich et. al |
| Chipewyan | 13 | Native American | Canadian | Reich et. al |
| Chono | 4 | Native American | Not used | Reich et. al |
| Colombian | 94 | Admixed | Not used | 1000 Genomes |
| Cree | 4 | Native American | Canadian | Reich et. al |
| Diaguita | 5 | Native American | Not used | Reich et. al |
| Embera | 5 | Native American | Not used | Reich et. al |
| Esan | 99 | African | Not used | 1000 Genomes |
| Finnish | 99 | European | Northern European | 1000 Genomes |
| French | 28 | European | Western European | HGDP |
| French, Jewish | 6 | European | Sephardic Jewish | Behar et. Al |
| British | 91 | European | Western European | 1000 Genomes |
| Guahibo | 6 | Native American | Not used | Reich et. al |
| Guarani | 6 | Native American | Not used | Reich et. al |
| Guaymi | 5 | Native American | Not used | Reich et. al |
| Gambian | 113 | African | Not used | 1000 Genomes |
| Han | 44 | Asian | Not used | HGDP |
| HRS, Self-identified African American | 2,502 | Admixed | Admixed | HRS |
| HRS, Other | 12,952 | Admixed | Admixed | HRS |
| Hulliche | 4 | Native American | Not used | Reich et. al |
| Spanish | 107 | European | Spanish | 1000 Genomes |
| Inga | 9 | Native American | Not used | Reich et. al |
| Kaqchikel | 13 | Native American | Central American | Reich et. al |
| Karitiana | 14 | Native American | Not used | HGDP |
| Kogi | 2 | Native American | Not used | Reich et. al |
| Luhya | 97 | African | Not used | 1000 Genomes |
| Mixe | 17 | Native American | Southern Mexican | Reich et. al |
| Mixtec | 5 | Native American | Southern Mexican | Reich et. al |
| Mongola | 10 | Asian | Not used | HGDP |
| Moroccan, Jewish | 15 | European | Sephardic Jewish | Behar et. Al |
| Mende | 85 | African | Not used | 1000 Genomes |
| Mexican | 64 | Admixed | Admixed | 1000 Genomes |
| Naukan | 16 | Asian | Not used | Reich et. al |
| Ojibwa | 5 | Native American | Canadian | Reich et. al |
| Orcadian | 15 | European | Western European | HGDP |
| Palikur | 3 | Native American | Not used | Reich et. al |
| Peruvian | 85 | Admixed | Admixed | 1000 Genomes |
| Piapoco | 7 | Native American | Amazonian | Reich et. al |
| Pima | 14 | Native American | Northern Mexican | HGDP |
| Puerto Rican | 104 | Admixed | Admixed | 1000 Genomes |
| Quechua | 40 | Native American | Not used | Reich et. al |
| Russian | 25 | European | Northern European | HGDP |
| Sardinian | 28 | European | Southern European | HGDP |
| Sephardic Jewish, Turkey | 10 | European | Sephardic Jewish | Behar et. Al |
| Sephardic Jewish, | 9 | European | Sephardic Jewish | Behar et. Al |
| Surui | 8 | Native American | Not used | HGDP |
| Tepehuano | 25 | Native American | Northern Mexican | Reich et. al |
| Teribe | 3 | Native American | Not used | Reich et. al |
| Ticuna | 6 | Native American | Amazonian | Reich et. al |
| Toba | 4 | Native American | Not used | Reich et. al |
| Tuscan | 107 | European | Southern European | 1000 Genomes |
| Waunana | 3 | Native American | Not used | Reich et. al |
| Wayuu | 11 | Native American | Not used | Reich et. al |
| Wichi | 5 | Native American | Not used | Reich et. al |
| Yaghan | 4 | Native American | Not used | HGDP |
| Yakut | 25 | Asian | Not used | HGDP |
| Yoruba | 108 | African | Not used | 1000 Genomes |
