# Supplementary material for: Native American admixture recapitulates population-specific migration and settlement of the continental United States
Source: PLoS Genet. 2019 Sep 23;15(9):e1008225. doi: 10.1371/journal.pgen.1008225 (PMC6756731; doi:10.1371/journal.pgen.1008225)
Supplement: S2 Table — A support vector machine (SVM) was created to characterize the European ancestry of individuals using ADMIXTURE values generated using masked European genotypes. 10-fold cross-validation was used to evaluate the performance of the SVM. Values shown are the numbers of individuals assigned to each ancestry in the validation procedure. Correct assignments are on the diagonal. (DOCX) [file pgen.1008225.s003.docx]

|  |  | Predicted ancestry | | | | |
| --- | --- | --- | --- | --- | --- | --- |
|  |  | Western | Northern | Southern | Spanish | Unassigned |
| Actual ancestry | Western | 128 | 0 | 0 | 2 | 4 |
|  | Northern | 0 | 123 | 0 | 0 | 1 |
|  | Southern | 0 | 0 | 129 | 0 | 6 |
|  | Spanish | 1 | 0 | 1 | 266 | 7 |
