# Supplementary material for: Native American admixture recapitulates population-specific migration and settlement of the continental United States
Source: PLoS Genet. 2019 Sep 23;15(9):e1008225. doi: 10.1371/journal.pgen.1008225 (PMC6756731; doi:10.1371/journal.pgen.1008225)
Supplement: S3 Table — The SVM was used to characterize New World individuals with known European ancestry. Values shown are the number of individuals assigned each European ancestry from each population. For each population, the known European ancestry is listed in the parentheses. (DOCX) [file pgen.1008225.s004.docx]

|  |  | Predicted ancestry | | | | |
| --- | --- | --- | --- | --- | --- | --- |
|  |  | Western | Northern | Southern | Spanish | Unassigned |
| 1KGP population | CEU (Western) | 98 | 1 | 0 | 0 | 0 |
|  | ASW (Western) | 49 | 0 | 0 | 0 | 2 |
|  | CLM (Spanish) | 0 | 0 | 0 | 94 | 0 |
|  | PEL (Spanish) | 0 | 0 | 3 | 69 | 13 |
