# Supplementary material for: Native American admixture recapitulates population-specific migration and settlement of the continental United States
Source: PLoS Genet. 2019 Sep 23;15(9):e1008225. doi: 10.1371/journal.pgen.1008225 (PMC6756731; doi:10.1371/journal.pgen.1008225)
Supplement: S4 Table — Haplotype heterozygosities (HH) were found for both the ancestry-specific and genome and whole-genome for each of the admixed populations. (DOCX) [file pgen.1008225.s005.docx]

| Ancestry | Population haplotype heterozygosity | | | | | | | | | | | | | | | | | | | | |
| --- | --- | --- | --- | --- | --- | --- | --- | --- | --- | --- | --- | --- | --- | --- | --- | --- | --- | --- | --- | --- | --- |
|  | ENC, AD | MA, AD | SE, AD | ESC, AD | WSC, AD | PAC, AD | WNC, WD | ENC, WD | SE, WD | WSC, WD | PAC, WD | MNT, WD | MNT, *Nuevomexicano* | MNT, Mexican | PAC, SD | WSC, SD | WNC, SD | SE, SD | Mexican, 1KGP | MA, SD | Puerto Rican, 1KGP |
| African | 0.965 | 0.964 | 0.964 | 0.964 | 0.964 | 0.965 |  |  |  |  |  |  | 0.964 | 0.966 | 0.965 | 0.964 | 0.907 | 0.965 | 0.964 | 0.963 | 0.962 |
| EuropWDn | 0.933 | 0.930 | 0.932 | 0.932 | 0.933 | 0.933 | 0.930 | 0.932 | 0.931 | 0.930 | 0.930 | 0.932 | 0.930 | 0.930 | 0.930 | 0.931 | 0.931 | 0.931 | 0.932 | 0.930 | 0.932 |
| Amerindian | 0.865 | 0.841 | 0.858 | 0.854 | 0.853 | 0.847 | 0.911 | 0.869 | 0.903 | 0.832 | 0.887 | 0.914 | 0.872 | 0.879 | 0.899 | 0.862 | 0.838 | 0.834 | 0.866 | 0.894 | 0.849 |
| All | 0.971 | 0.971 | 0.971 | 0.971 | 0.971 | 0.972 | 0.933 | 0.934 | 0.935 | 0.932 | 0.932 | 0.934 | 0.937 | 0.938 | 0.941 | 0.942 | 0.938 | 0.962 | 0.939 | 0.945 | 0.943 |

**S4 Table. Haplotype heterozygosities of ancestry-specific and whole genome haplotypes.** Haplotype heterozygosity was found for the ancestry-specific (African, EuropWDn, and Native American) genomes and whole-genome (All)
