# Supplementary figures and images for: Native American admixture recapitulates population-specific migration and settlement of the continental United States
Source: PLoS Genet. 2019 Sep 23;15(9):e1008225. doi: 10.1371/journal.pgen.1008225 (PMC6756731; doi:10.1371/journal.pgen.1008225)

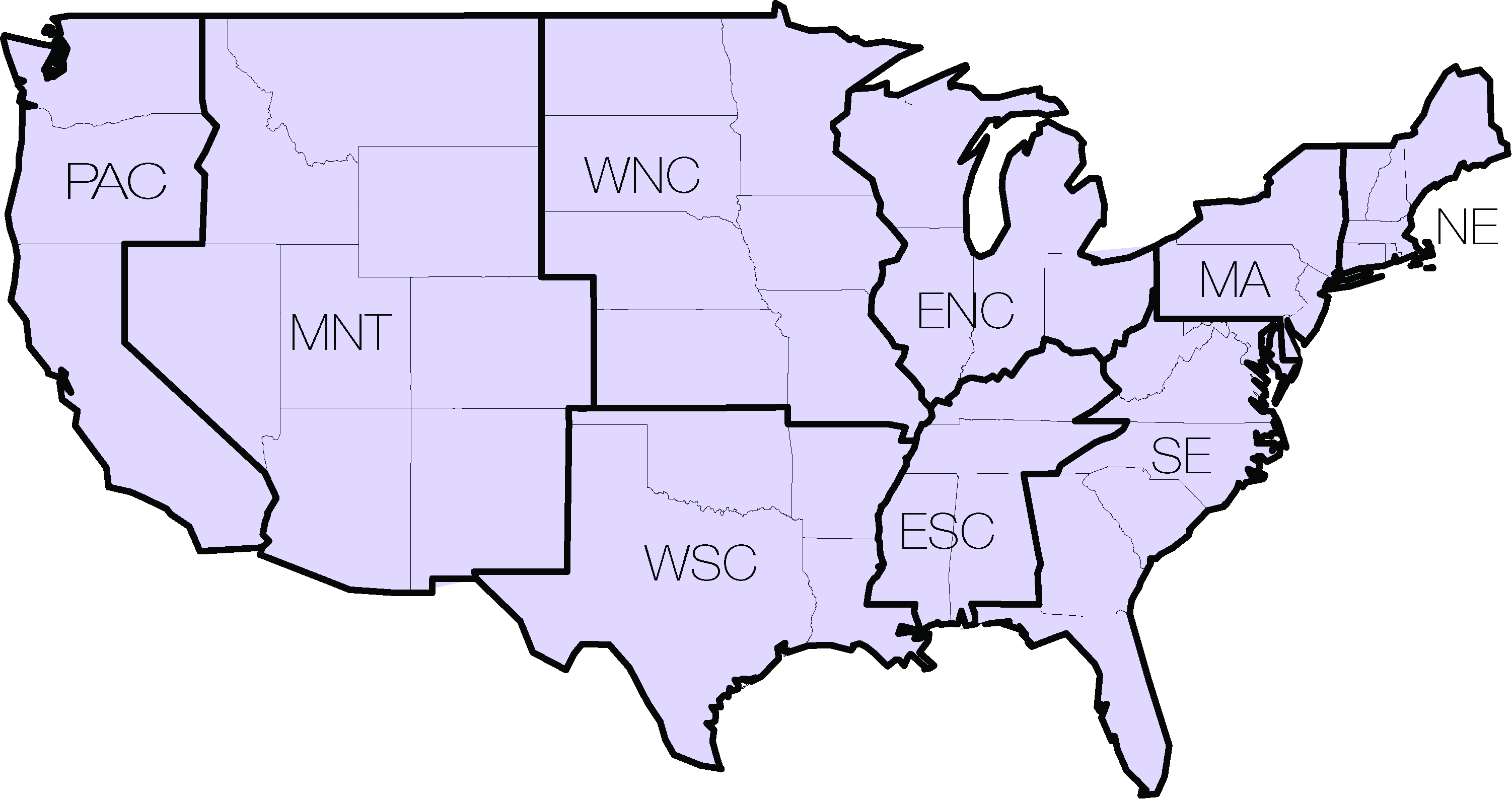

Supplement: S1 Fig — The census regions, in semi-clockwise order, are: West North-Central (WNC), East North-Central (ENC), Northeast (NE), Mid-Atlantic (MA), Southeast (SE), East South-Central (ESC), West South-Central (WSC), Mountain (MNT), and Pacific (PAC). (TIF) [file pgen.1008225.s006.tif]

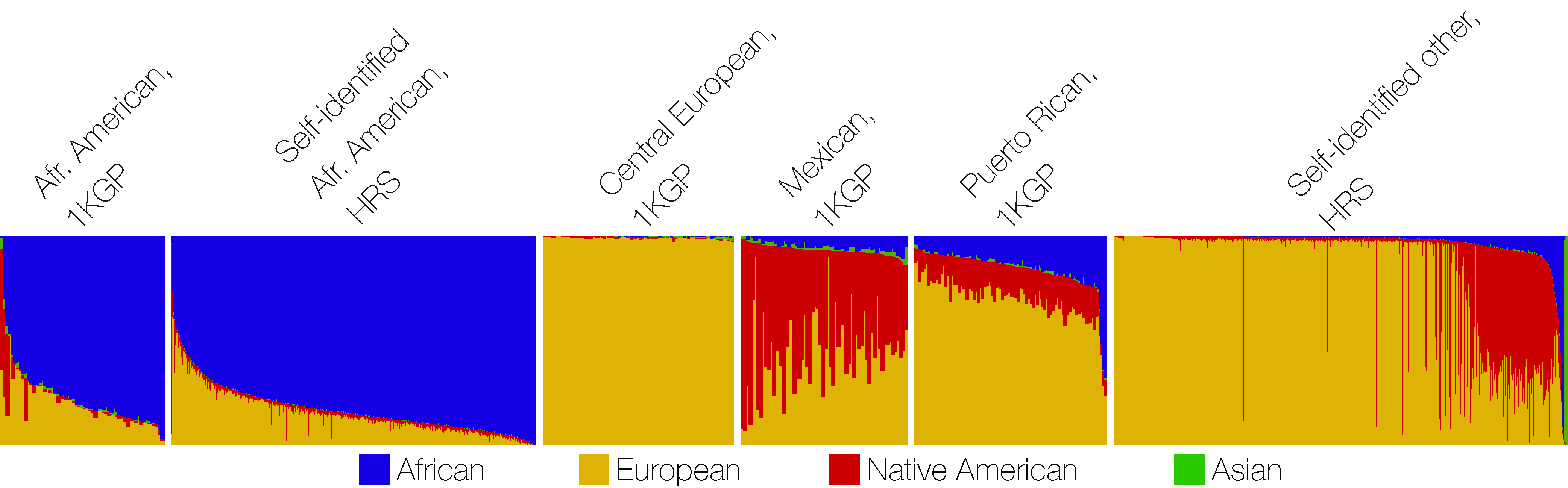

Supplement: S2 Fig — African (blue), European (yellow), Native American (red), and East Asian (green) ancestry components are shown. The HRS individuals were divided into two groups based on their self-identified status as African-Americans and all others. HRS individuals shown in comparison to African (African American–ASW), European (Central European–CEU), Latin American (Mexican–MXL and Puerto Rican–PUR) 1KGP reference populations. (TIF) [file pgen.1008225.s007.tif]

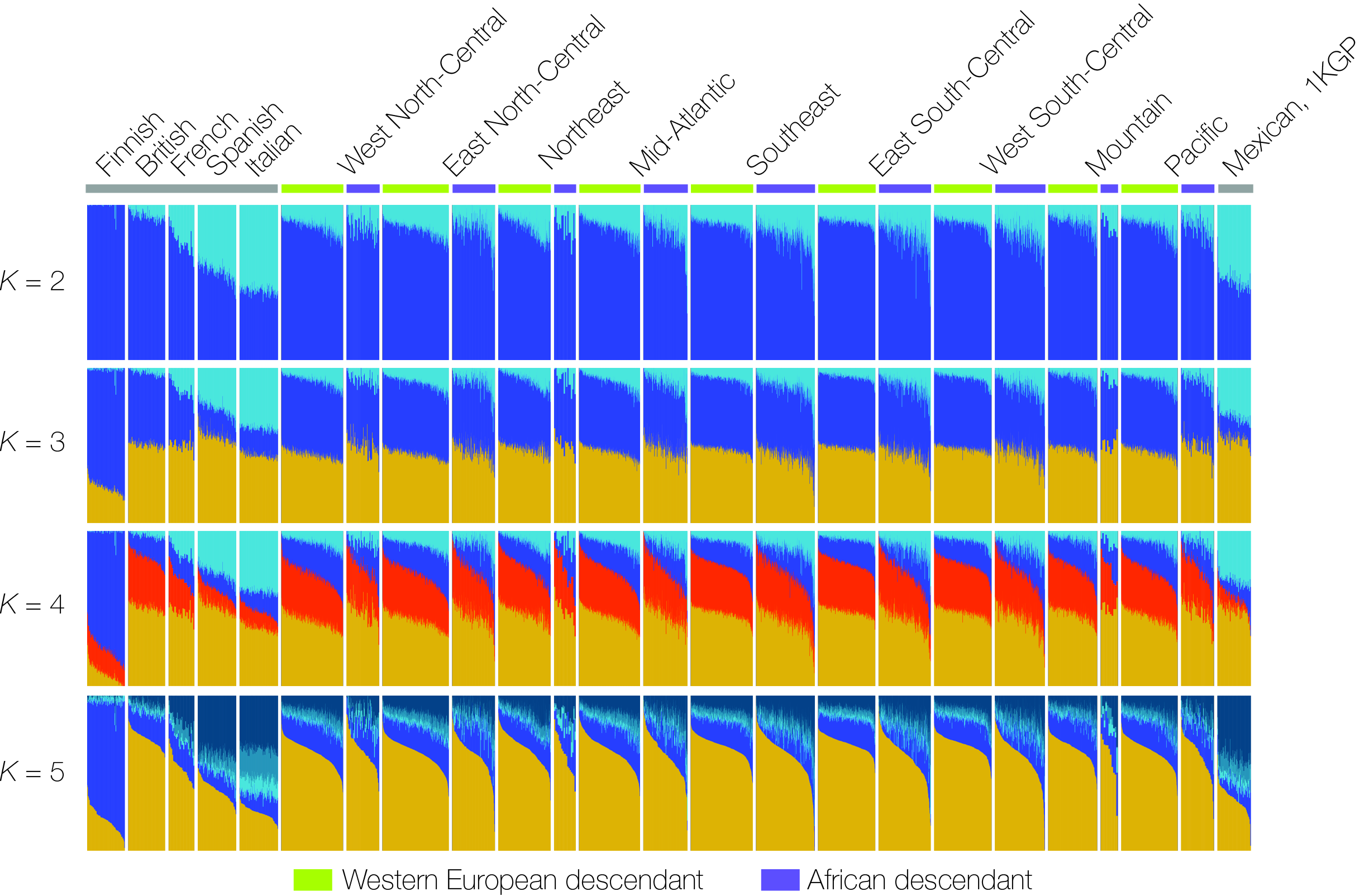

Supplement: S3 Fig — Individuals were placed into genetic ancestry groups using the SVM classifier as described in the Materials and Methods. ADMIXTURE was run and HRS individuals from the two genetic ancestry groups (AD-purple and WD-lime) are plotted according to their geographic region of origin along with individuals from European (Finnish, British, French, Spanish, and Italian) and Mexican 1KGP reference populations. ADMIXTURE was run using k = 2, 3, 4, and 5 populations. (TIF) [file pgen.1008225.s008.tif]

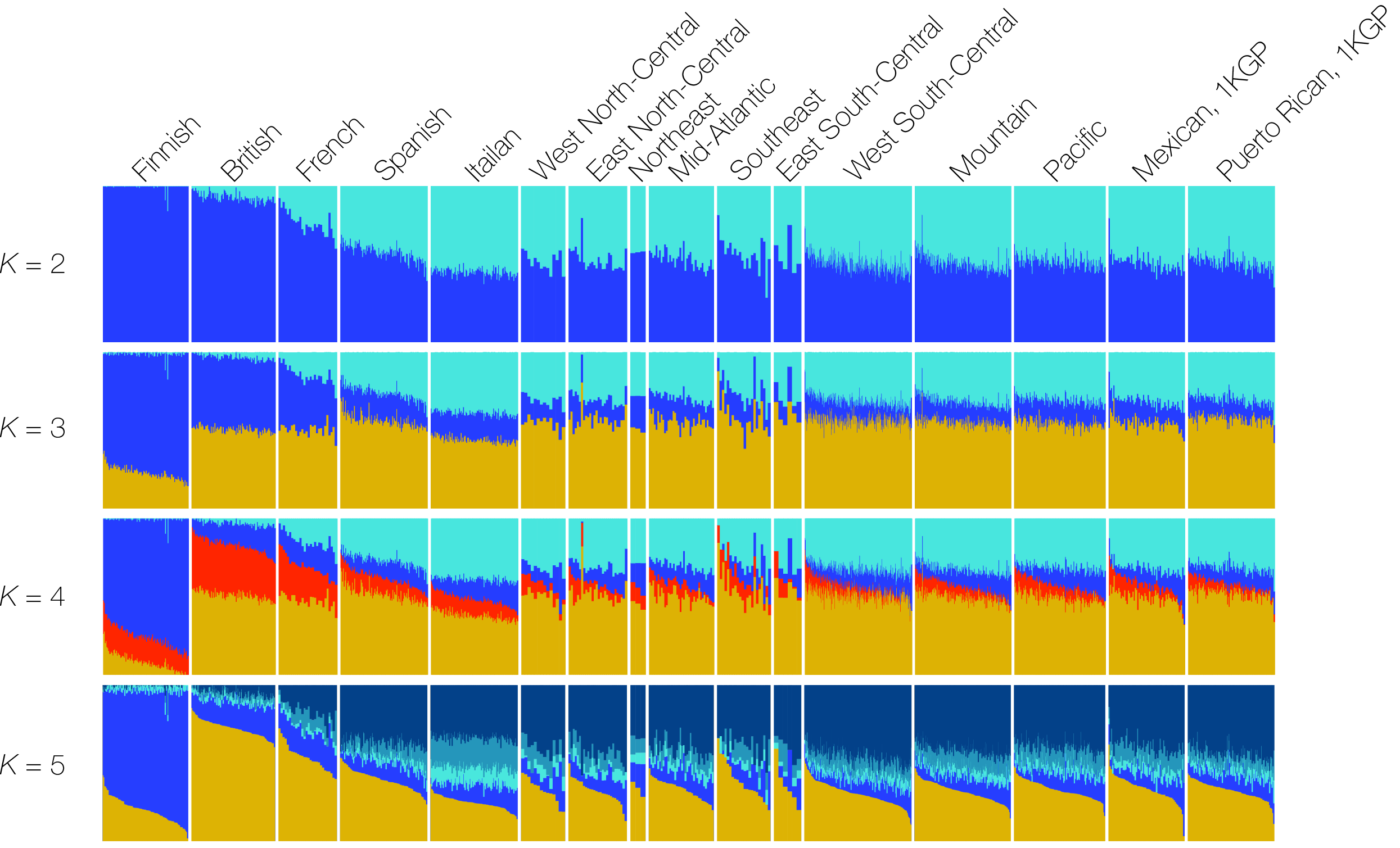

Supplement: S4 Fig — Individuals were placed into genetic ancestry groups using the SVM classifier as described in the Materials and Methods. ADMIXTURE was run and HRS individuals from the Spanish descendant group are plotted according to their geographic region of origin along with individuals from European (Finnish, British, French, Spanish, and Italian), Mexican, and Puerto Rican 1KGP reference populations. ADMIXTURE was run using k = 2, 3, 4, and 5 populations. (TIF) [file pgen.1008225.s009.tif]

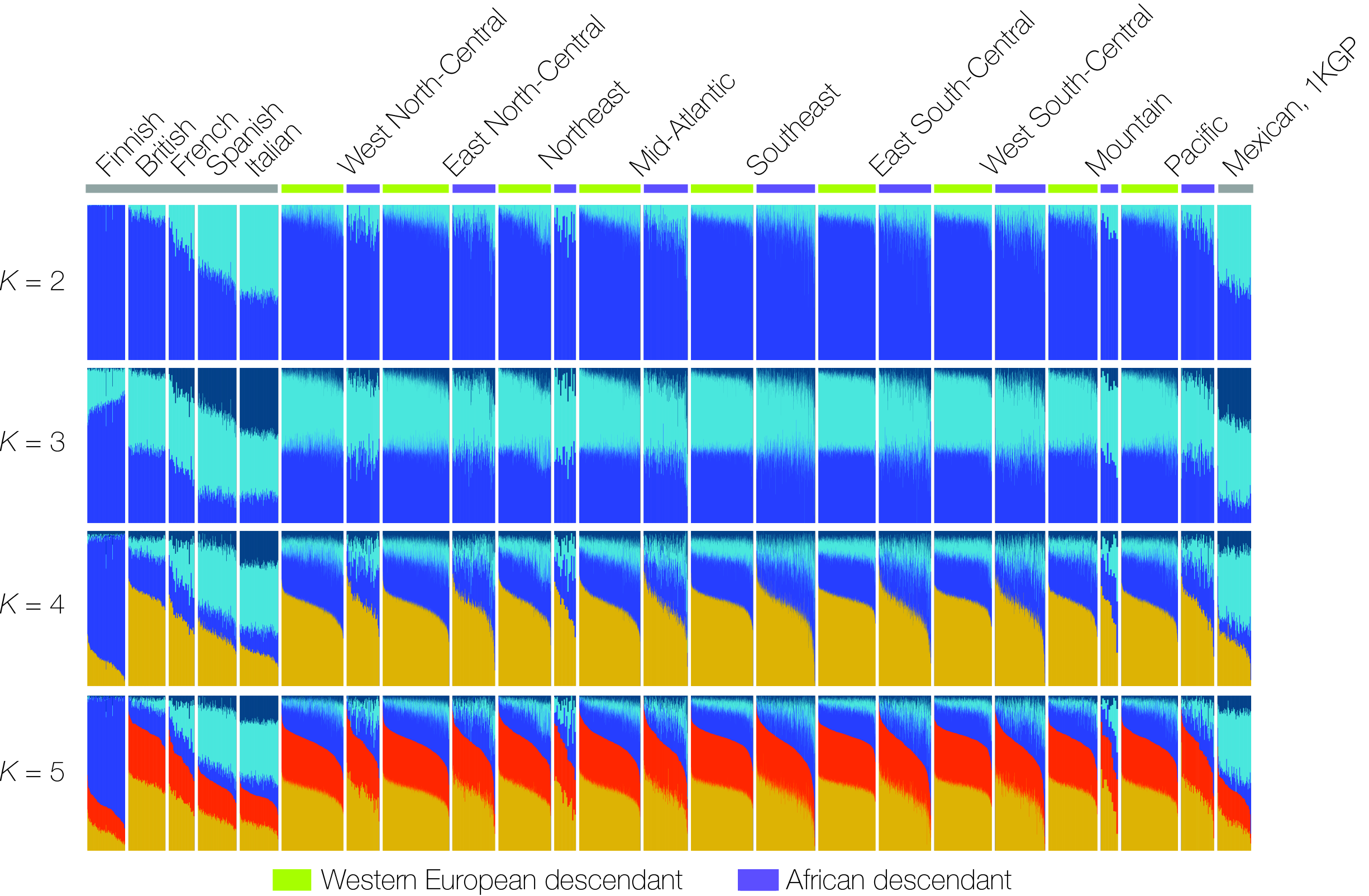

Supplement: S5 Fig — ADMIXTURE was run on masked European haplotypes from WD and AD individuals 20 times for K = 2 to K = 5, using different seeds for each run. The CLUMPP utility was used to identify corresponding inferred ancestries across ADMIXTURE runs that used the same K. The means of the CLUMPP characterized ancestries are shown here. (TIF) [file pgen.1008225.s010.tif]

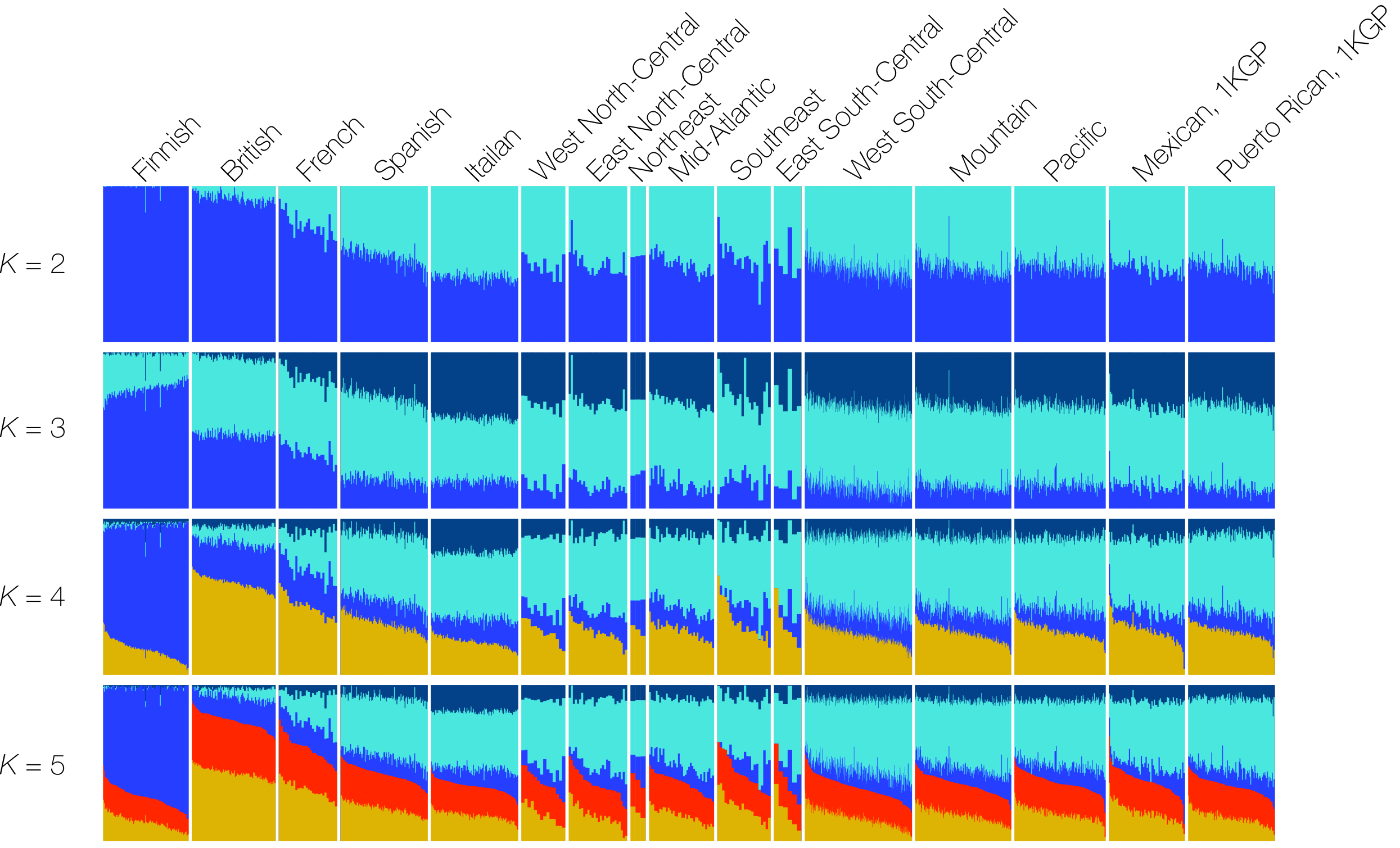

Supplement: S6 Fig — ADMIXTURE was run on masked European haplotypes from SD individuals 20 times for K = 2 to K = 5, using different seeds for each run. The CLUMPP utility was used to identify corresponding inferred ancestries across ADMIXTURE runs that used the same K. The means of the CLUMPP characterized ancestries are shown here. (TIF) [file pgen.1008225.s011.tif]

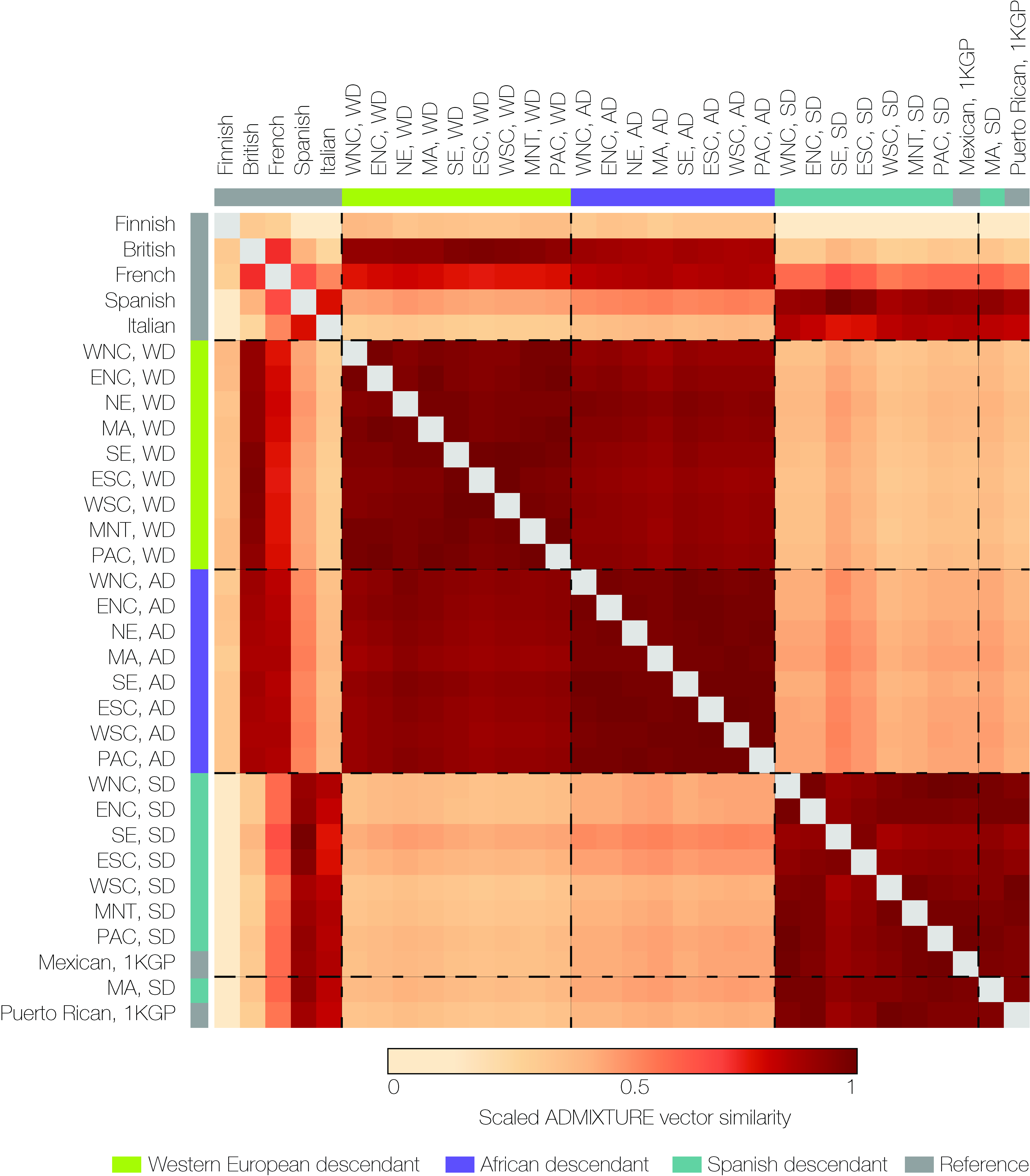

Supplement: S7 Fig — For each population, the mean CLUMPP-ADMIXTURE vector was found by concatenating the vectors from K = 2 to K = 5 and taking the mean of each component across individuals. The Euclidean distance in these vectors was found between all populations, and rescaled from 0 to 1. The similarity between populations was found as 1—the distance. (TIF) [file pgen.1008225.s012.tif]

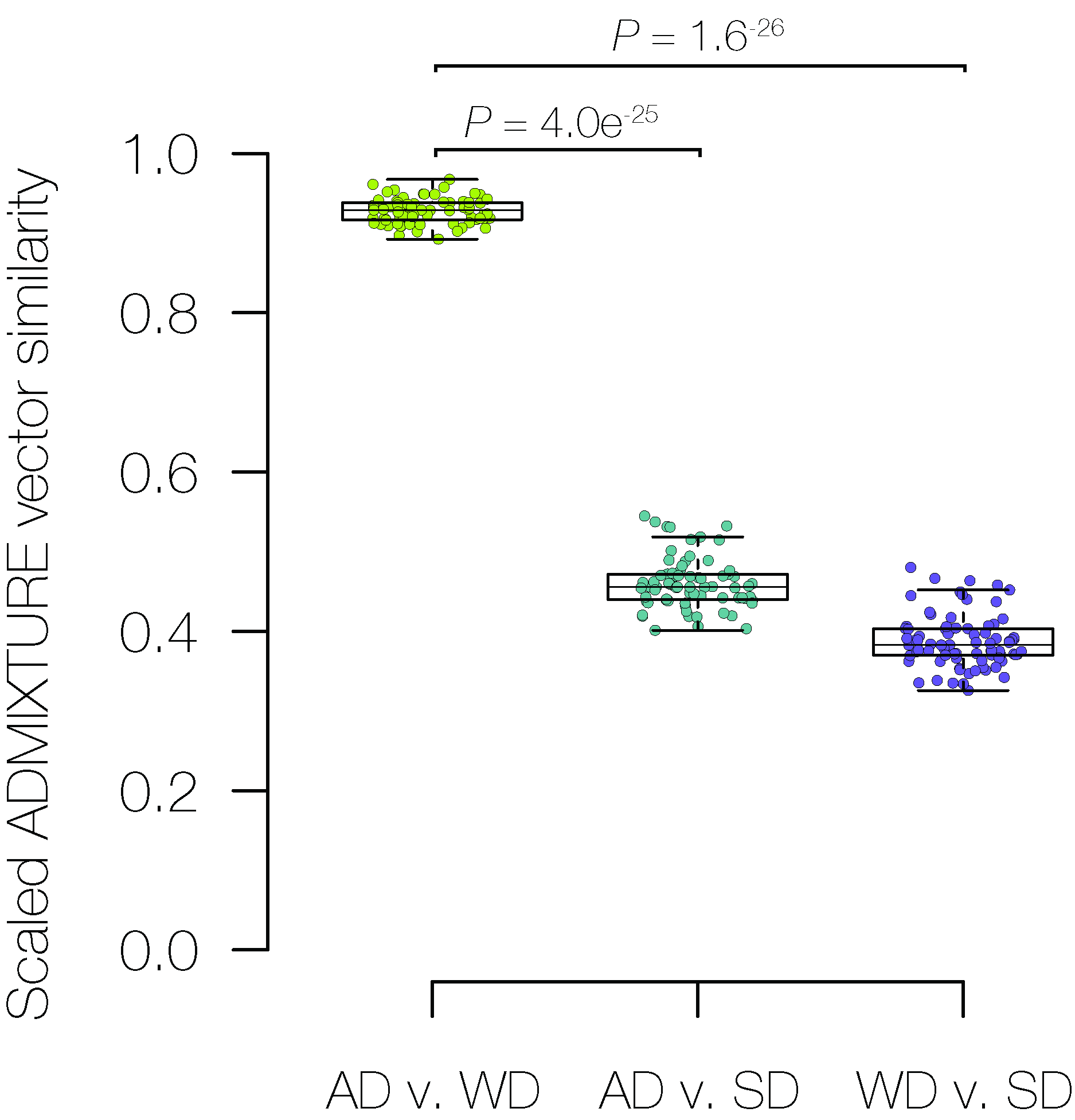

Supplement: S8 Fig — European CLUMPP-ADMIXTURE similarities were found between African descendant (AD), Western European descendant (WD), and Spanish descendant (SD) populations. Differences in similarity between two population groups were assessed using a Wilcoxon rank-sum test. (TIF) [file pgen.1008225.s013.tif]

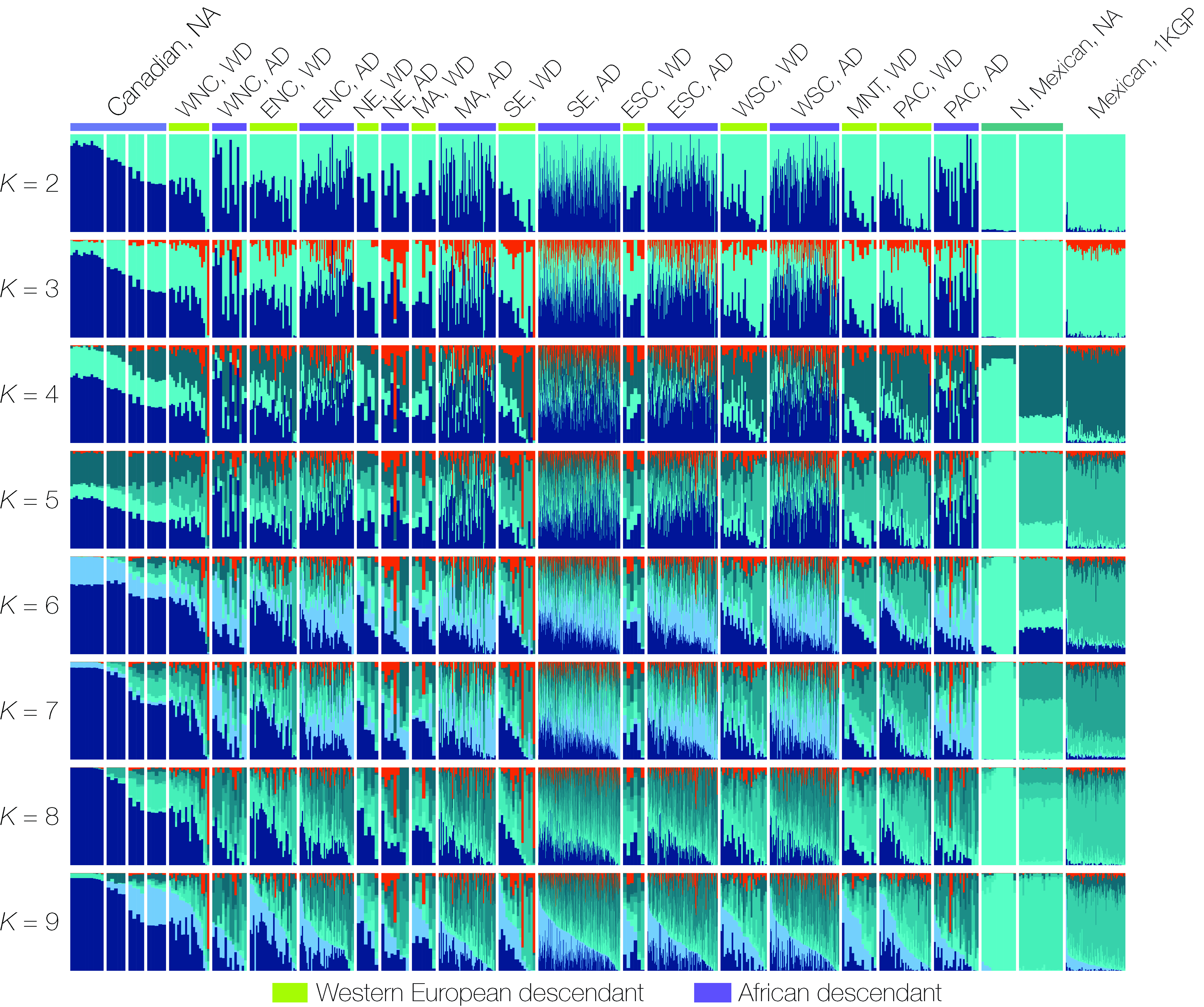

Supplement: S10 Fig — ADMIXTURE was run on masked Native American haplotypes from AD and WD individuals 20 times for K = 2 to K = 9, using different seeds for each run. The CLUMPP utility was used to identify corresponding inferred ancestries across ADMIXTURE runs that used the same K. The means of the CLUMPP characterized ancestries are shown here. (TIF) [file pgen.1008225.s015.tif]

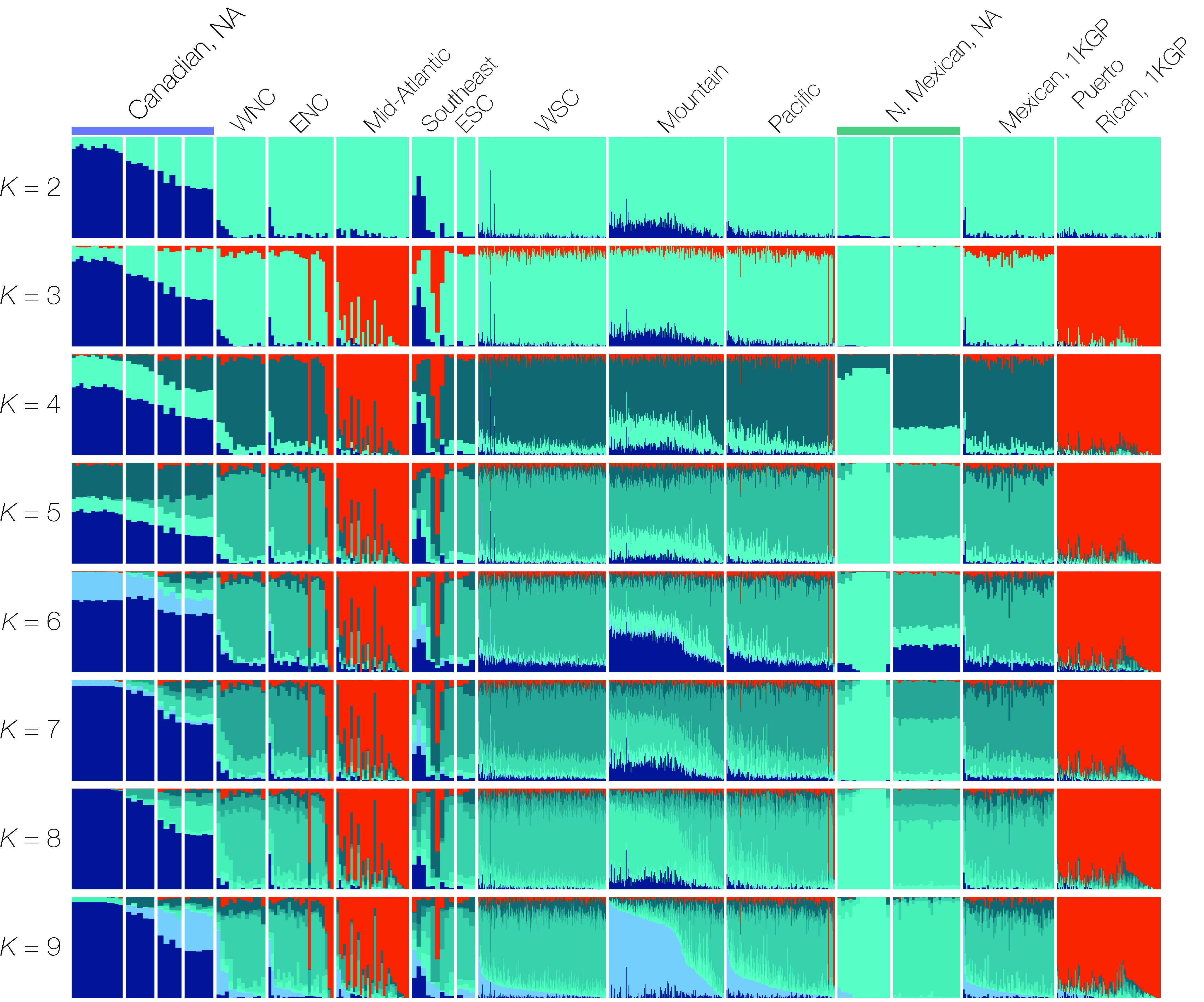

Supplement: S11 Fig — ADMIXTURE was run on masked Native American haplotypes from HL individuals 20 times for K = 2 to K = 9, using different seeds for each run. The CLUMPP utility was used to identify corresponding inferred ancestries across ADMIXTURE runs that used the same K. The means of the CLUMPP characterized ancestries are shown here. (TIF) [file pgen.1008225.s016.tif]

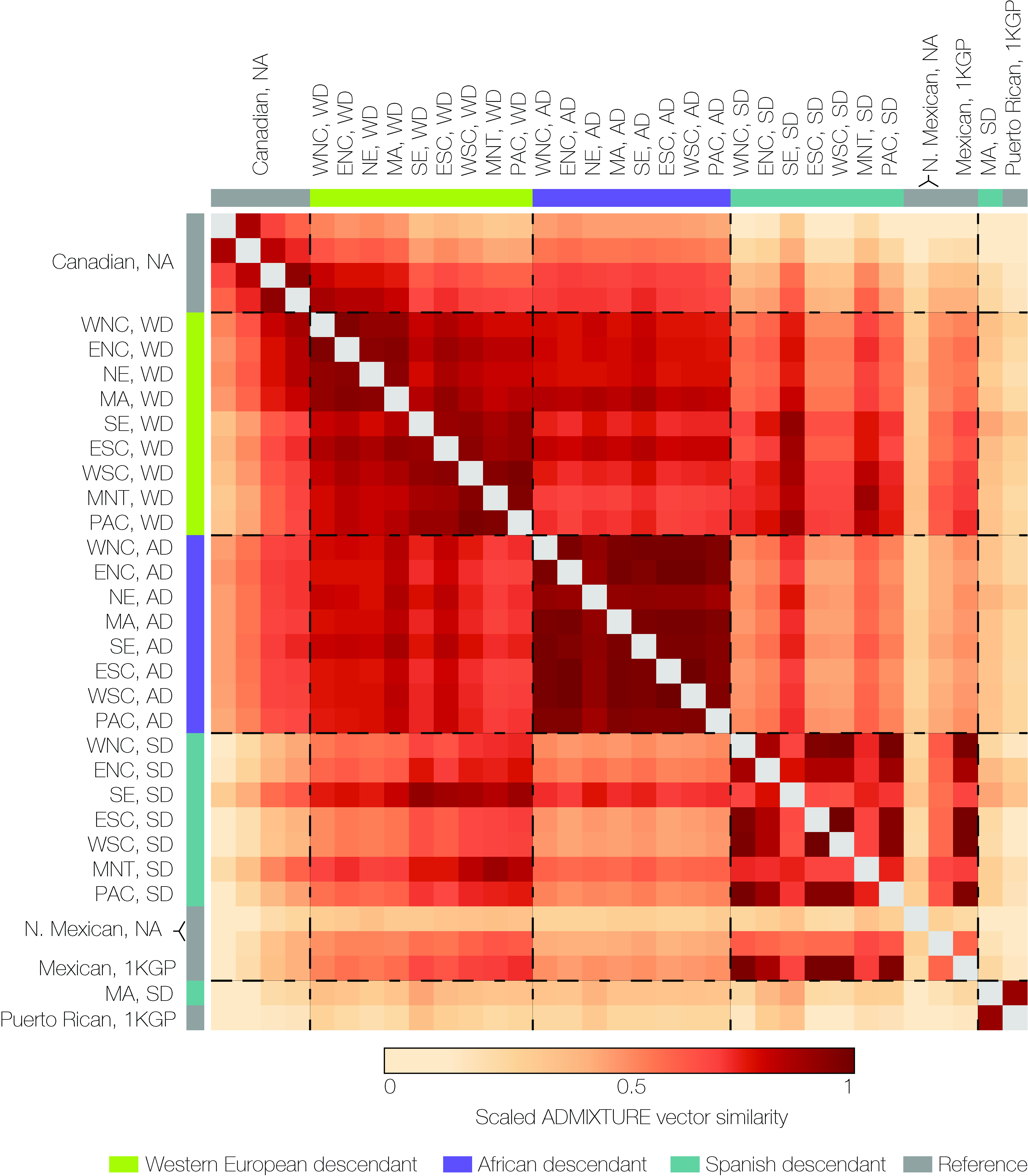

Supplement: S12 Fig — For each population, the mean CLUMPP-ADMIXTURE vector was found by concatenating the vectors from K = 2 to K = 9 and taking the mean of each component across individuals. The Euclidean distance in these vectors was found between all populations, and rescaled from 0 to 1. The similarity between populations was found as 1—the distance. (TIF) [file pgen.1008225.s017.tif]

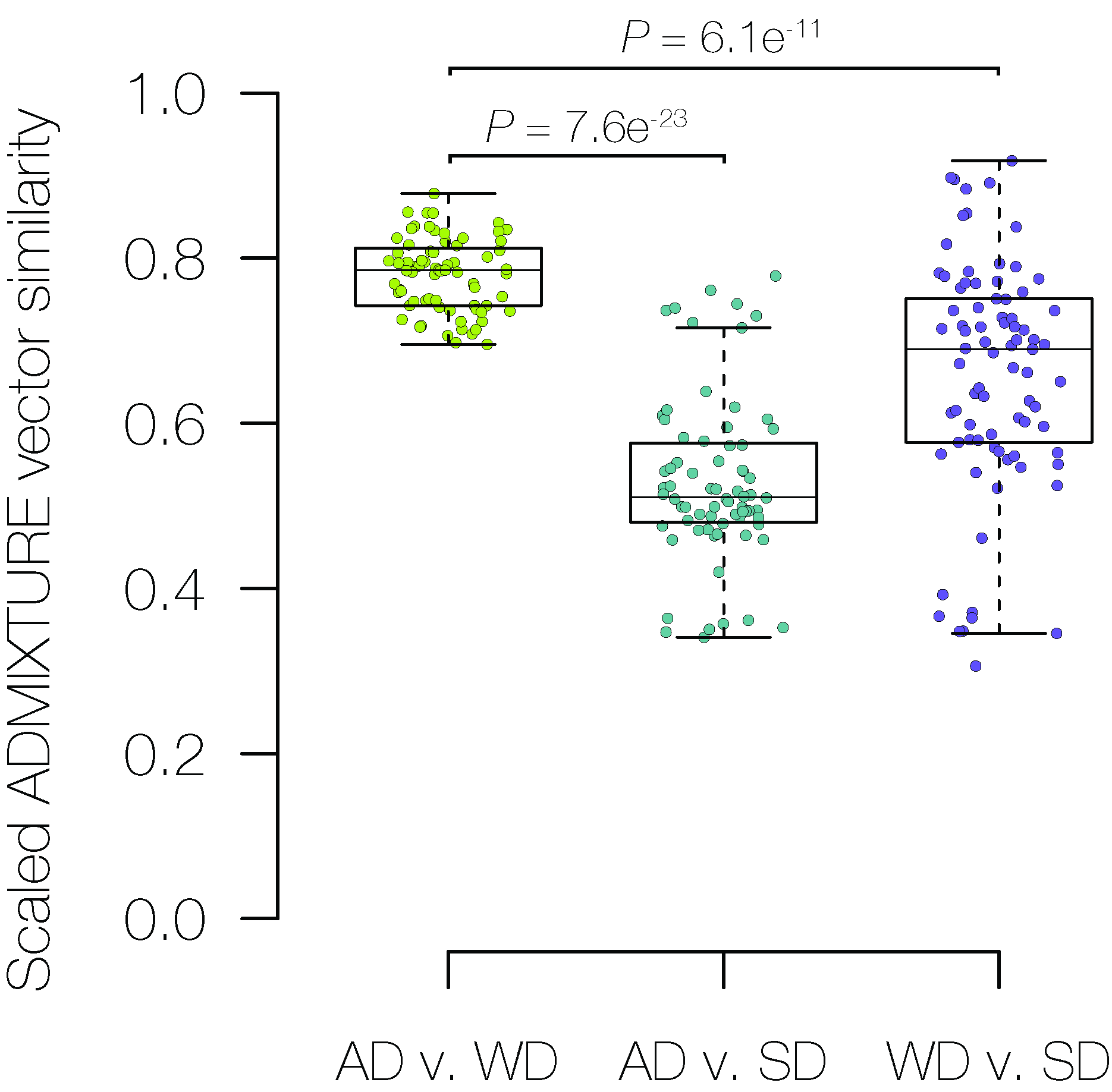

Supplement: S13 Fig — Native American CLUMPP-ADMIXTURE similarities were found between African descendant (AD), Western European descendant (WD), and Spanish descendant (SD) populations. Differences in similarity between two population groups were assessed using a Wilcoxon rank-sum test. (TIF) [file pgen.1008225.s018.tif]

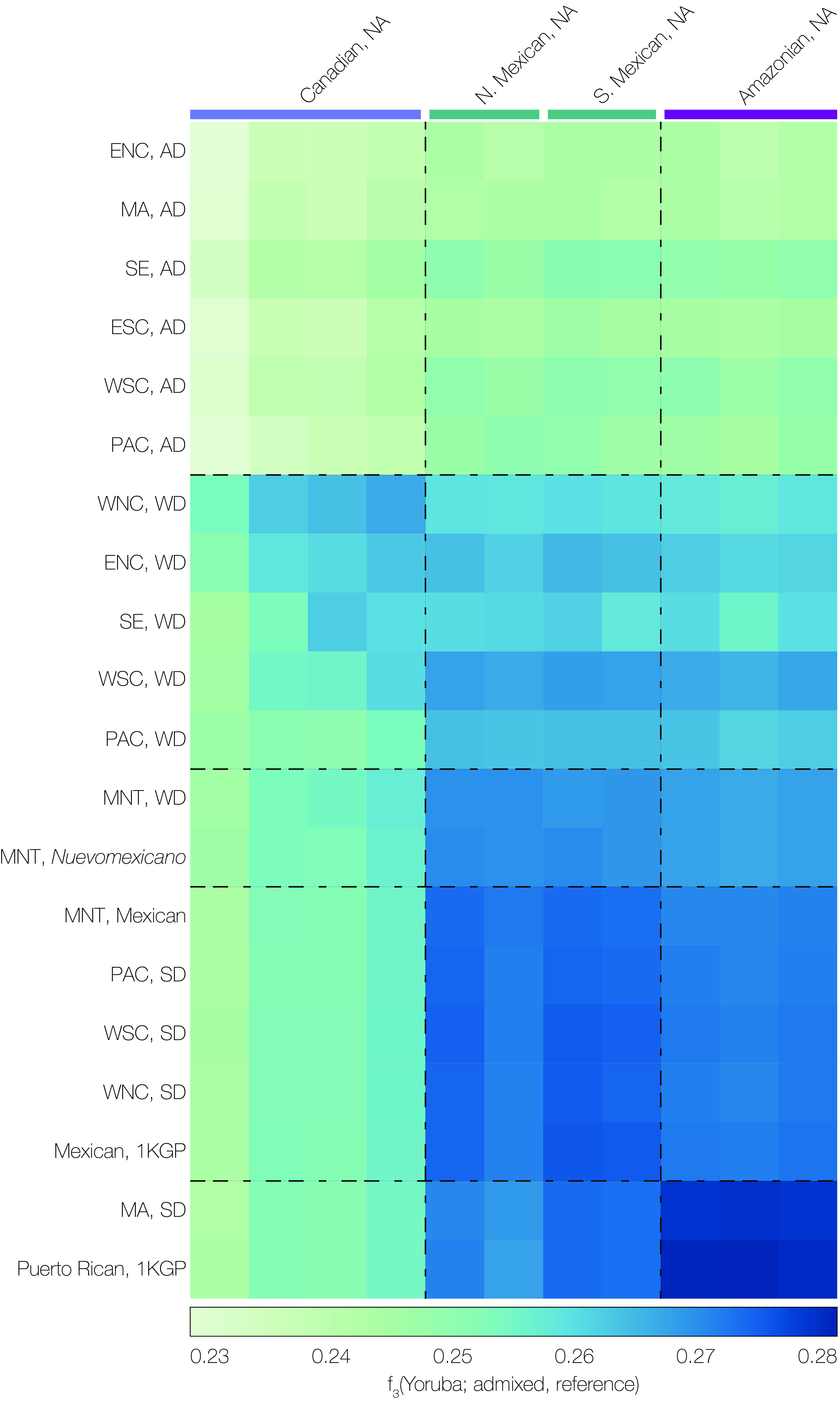

Supplement: S14 Fig — Masked Native American genotypes were used to compare admixed US populations to Native American reference populations using the outgroup f3 statistic. The f3-statistics were computed as f3(YRI; admixed, reference). (TIF) [file pgen.1008225.s019.tif]

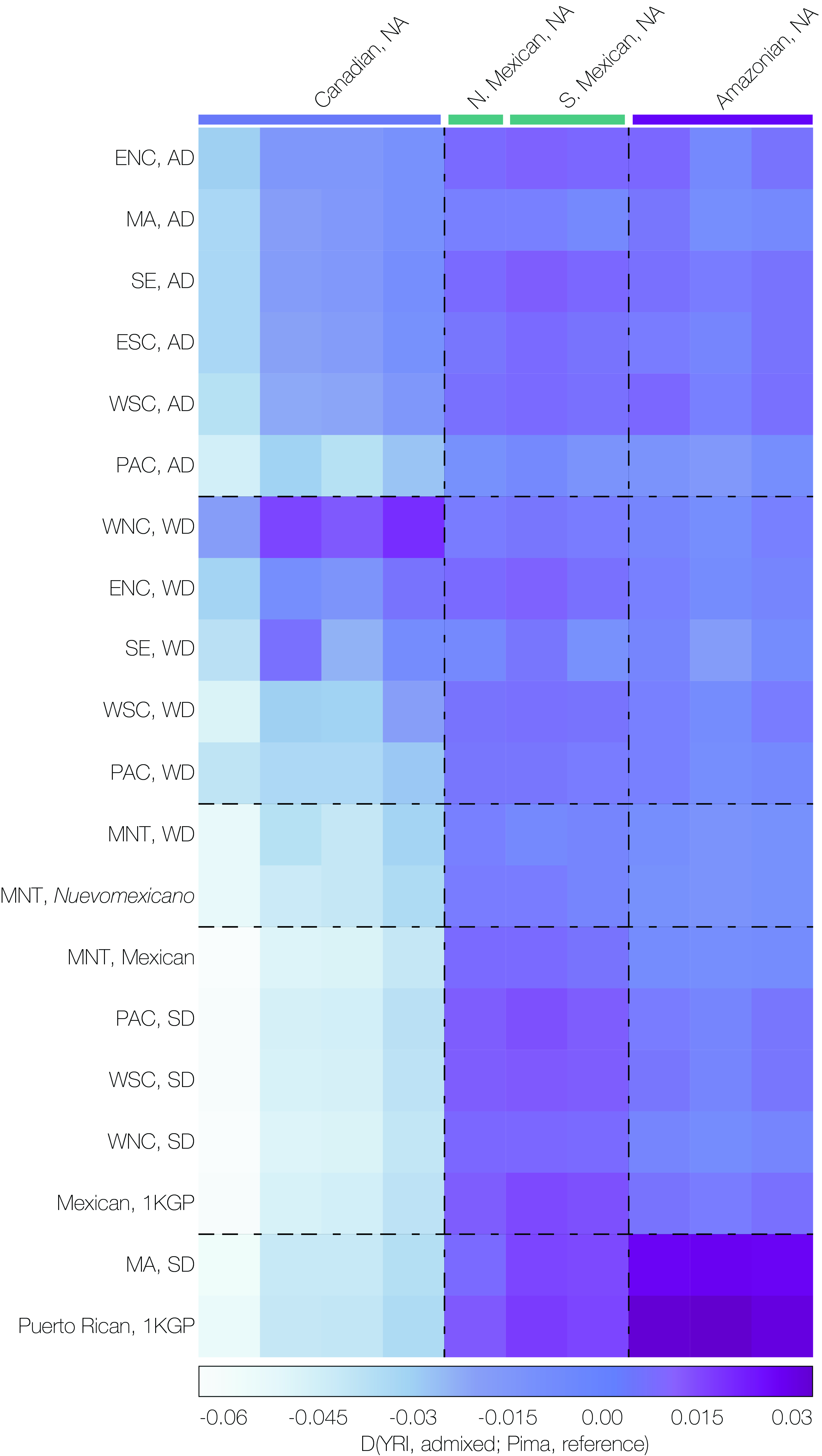

Supplement: S15 Fig — Masked Native American genotypes were used to compare admixed US populations to Native American reference populations using the D statistic. The D-statistics were computed as D(YRI, admixed; Pima, reference) to determine whether admixed populations were more closely related to the modern Pima population or to another Native American population. (TIF) [file pgen.1008225.s020.tif]

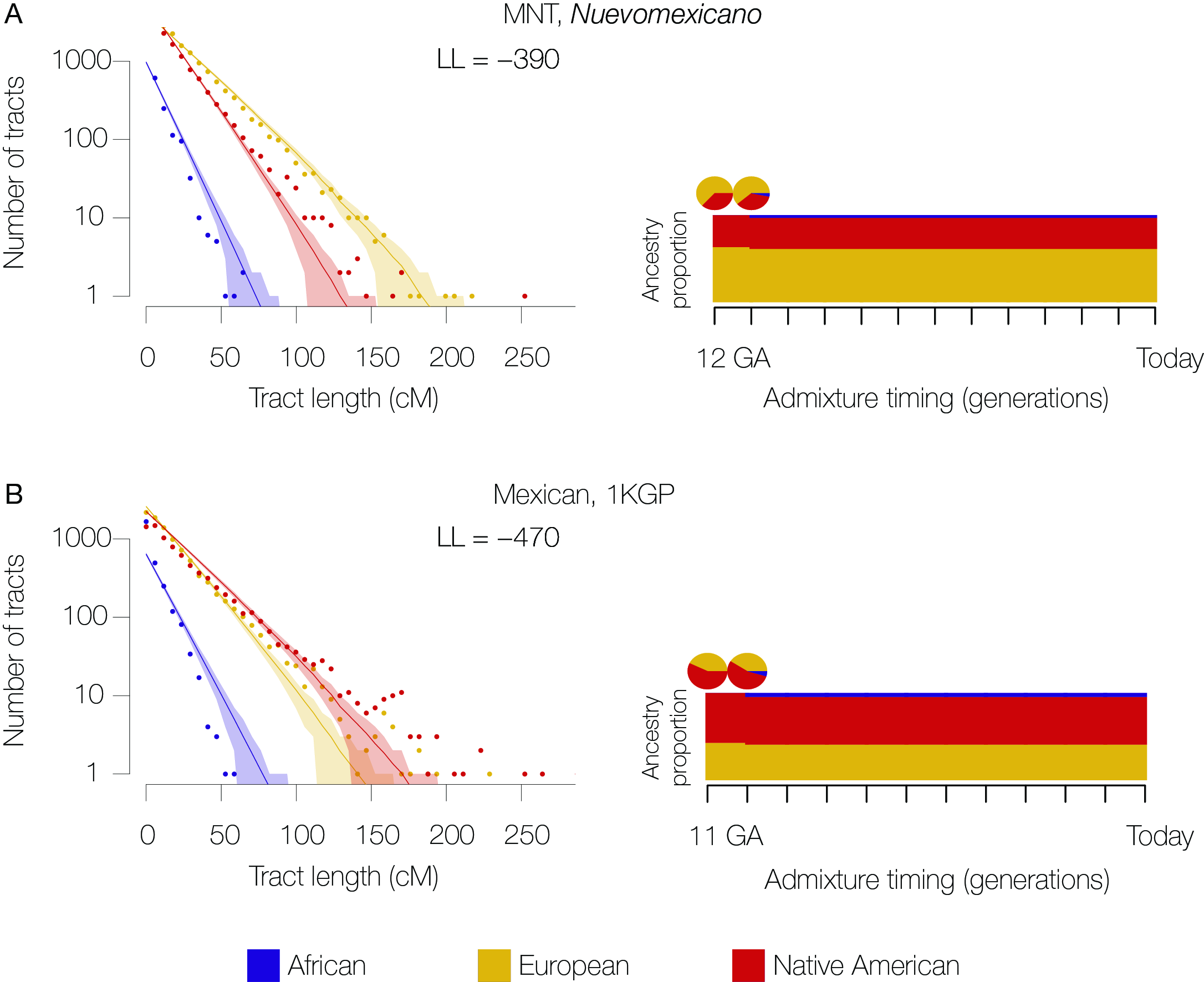

Supplement: S16 Fig — (Left side) Observed (points) and predicted (solid line) ancestry tract size distributions; the shaded areas represent 95% confidence intervals. (Right side) Admixture event timings are shown together with ancestry proportions. Each inferred admixture event is indicated by a circle, which is scaled according to the size of the contribution to the population and also shows the relative ancestry proportions. The y-axes of the charts show the inferred continental ancestry fractions, and the x-axes show time as the number of generations ago (GA). (A) Inferred admixture timing in the MNT Nuevomexicano population. (B) Inferred admixture timing in the 1KGP Mexican population. (TIF) [file pgen.1008225.s021.tif]

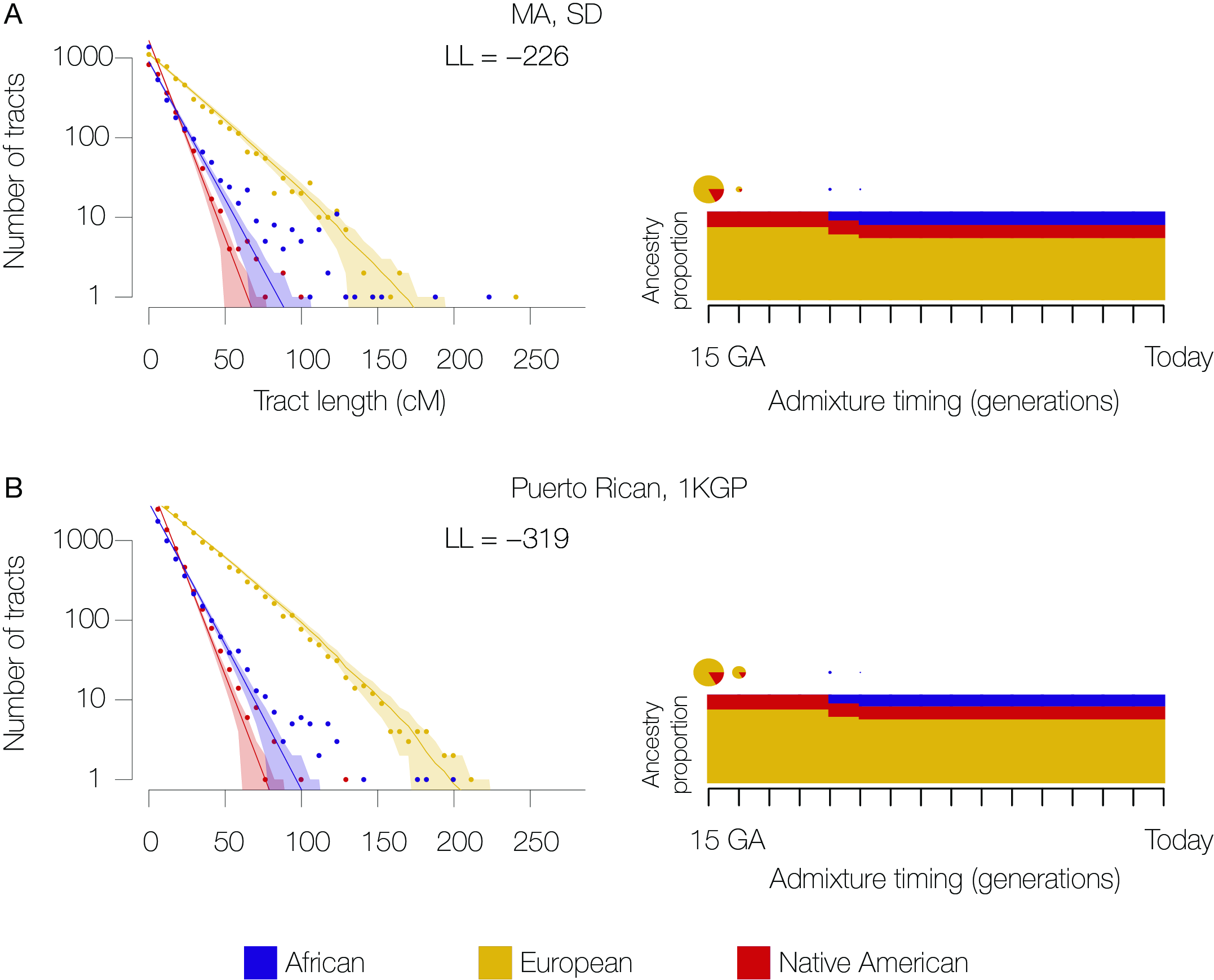

Supplement: S17 Fig — (Left side) Observed (points) and predicted (solid line) ancestry tract size distributions; the shaded areas represent 95% confidence intervals. (Right side) Admixture event timings are shown together with ancestry proportions. Each inferred admixture event is indicated by a circle, which is scaled according to the size of the contribution to the population and also shows the relative ancestry proportions. The y-axes of the charts show the inferred continental ancestry fractions, and the x-axes show time as the number of generations ago (GA). (A) Inferred admixture timing in the Mid-Atlantic Spanish descendant population. (B) Inferred admixture timing in the 1KGP Puerto Rican population. (TIF) [file pgen.1008225.s022.tif]

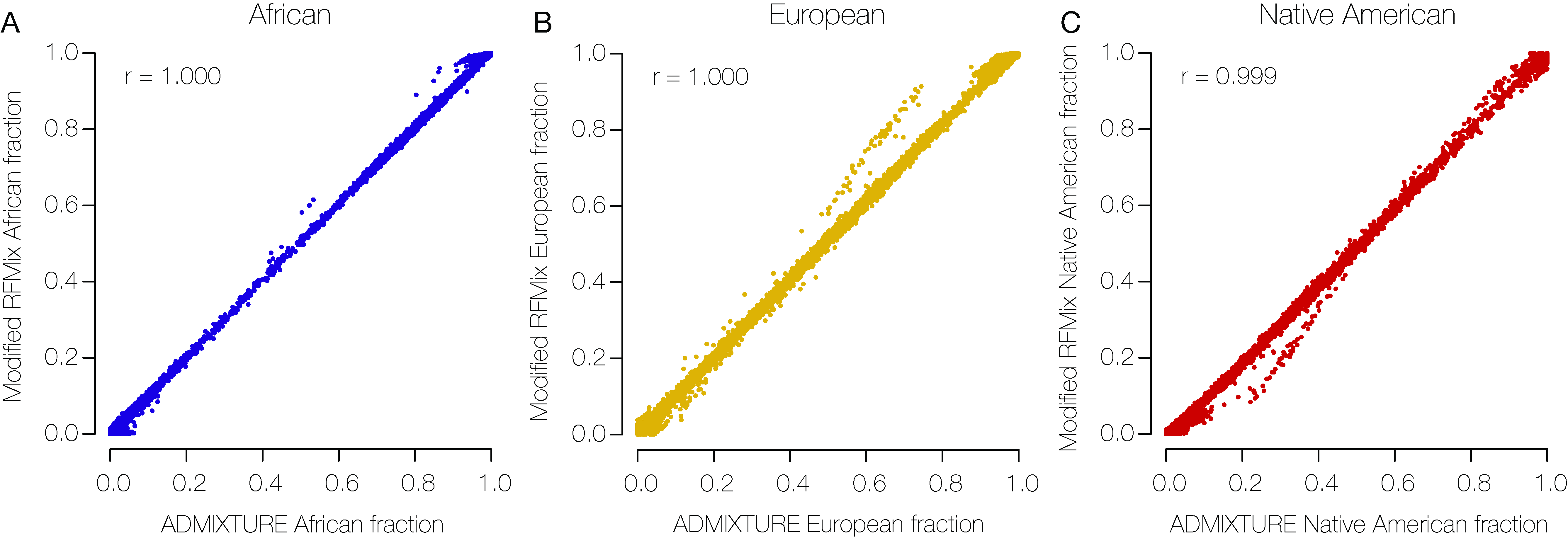

Supplement: S18 Fig — The RFMix utility was modified to reduce the computational time. The resulting genome-wide continental ancestry fractions (as determined by summing the local ancestry across each individual) were compared to estimates given by ADMIXTURE for (A) African ancestry, (B) European ancestry, and (C) Native American ancestry. All correlations between RFMix and ADMIXTURE continental ancestry fractions were significant (p < 1e-10, Pearson linear correlation). (TIF) [file pgen.1008225.s023.tif]

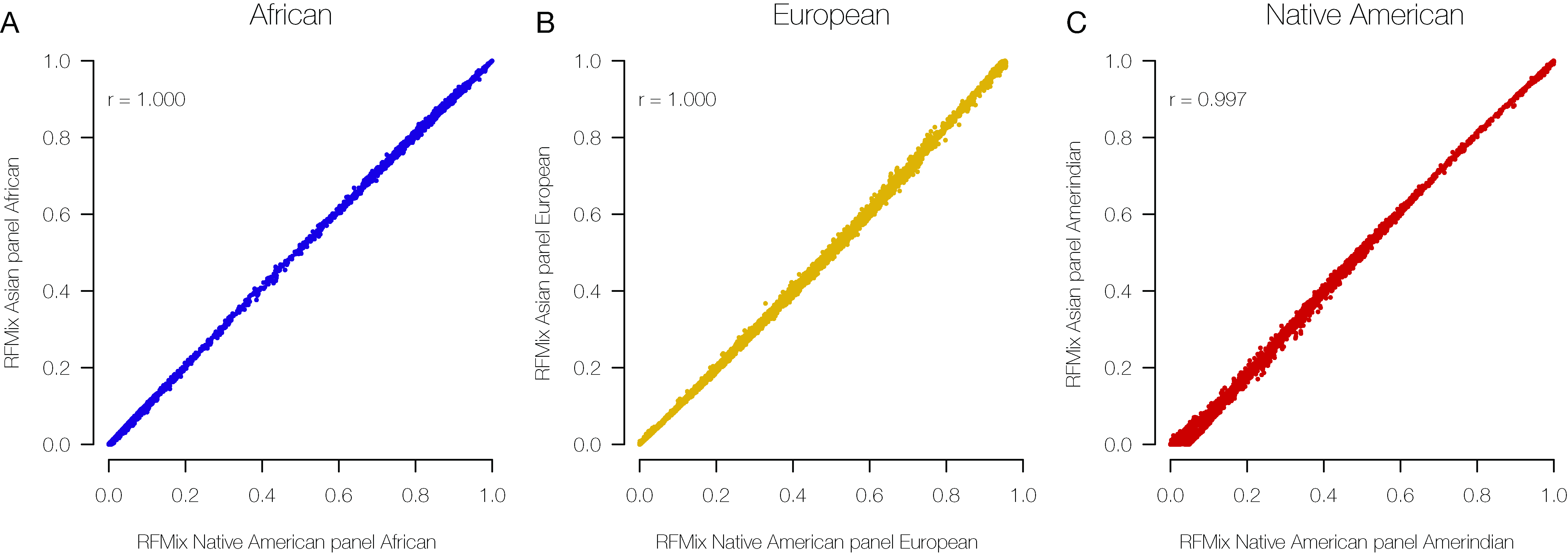

Supplement: S19 Fig — The modified RFMix utility was used to infer local ancestry as above in S18 Fig, however, modern East Asian populations were used as the reference for Native American ancestry rather than Native American populations. The genome-wide continental ancestry fractions were compared between this East Asian reference population analysis and those generated using Native American reference populations for (A) African ancestry, (B) European ancestry, and (C) Native American ancestry. All correlations of continental ancestry between the two RFMix analyses fractions were significant (p < 1e-10, Pearson linear correlation). (TIF) [file pgen.1008225.s024.tif]
